# Emergent topological quantum orbits in the charge density wave phase of kagome metal CsV_3_Sb_5_

**DOI:** 10.1038/s41535-023-00571-w

**Published:** 2023-07-28

**Authors:** Hengxin Tan, Yongkang Li, Yizhou Liu, Daniel Kaplan, Ziqiang Wang, Binghai Yan

**Affiliations:** 1 Department of Condensed Matter Physics, Weizmann Institute of Science, Rehovot, 7610001 Israel; 2 Department of Physics, Boston College, Chestnut Hill, MA 02467 USA

**Keywords:** Electronic properties and materials, Theory and computation

## Abstract

The recently discovered kagome materials *A*V_3_Sb_5_ (*A* = K, Rb, Cs) attract intense research interest in intertwined topology, superconductivity, and charge density waves (CDW). Although the in-plane 2 × 2 CDW is well studied, its out-of-plane structural correlation with the Fermi surface properties is less understood. In this work, we advance the theoretical description of quantum oscillations and investigate the Fermi surface properties in the three-dimensional CDW phase of CsV_3_Sb_5_. We derived Fermi-energy-resolved and layer-resolved quantum orbits that agree quantitatively with recent experiments in the fundamental frequency, cyclotron mass, and topology. We reveal a complex Dirac nodal network that would lead to a *π* Berry phase of a quantum orbit in the spinless case. However, the phase shift of topological quantum orbits is contributed by the orbital moment and Zeeman effect besides the Berry phase in the presence of spin-orbital coupling (SOC). Therefore, we can observe topological quantum orbits with a *π* phase shift in otherwise trivial orbits without SOC, contrary to common perception. Our work reveals the rich topological nature of kagome materials and paves a path to resolve different topological origins of quantum orbits.

## Introduction

The recently discovered kagome superconductors *A*V_3_Sb_5_ (*A* = K, Rb, Cs)^1^ stimulate extensive studies for their intriguing charge density waves (CDW)^2–10^, possible symmetry breaking without magnetism^2,11–21^, topological band structure^3,4,22–24^, and exotic superconductivity^5,25–27^. Aiming at deriving the Fermi surface (FS) property of CsV_3_Sb_5_, a dozen quantum oscillation experiments reveal complicated but similar oscillation frequencies^28–36^, bearing the Fermi energy variation in different samples. However, the nontrivial Fermi pockets (or quantum orbits), characterized by a *π* phase shift in the fundamental quantum oscillation, are dissimilar in different reports. For example, ref. ^30^ claimed nontrivial pockets are 73 and 727 T in the fundamental oscillation frequency. Chapai et al.^35^ found Fermi pockets of 79, 736, and 804 T are nontrivial. Broyles et al.^36^ reported 28, 74, and 85 T to have a *π* phase shift. Shrestha et al.^32^ stated that all seven quantum orbits (from 18 to 2135 T) observed are topological.

It is commonly believed that the measured *π* phase shift in a quantum oscillation originates from the *π* Berry phase (*ϕ*_*B*_) of Dirac fermion in the system^37,38^. For example, graphene exhibits the *π* Berry phase in quantum oscillation^39^. However, as pointed out by refs. ^40,41^, the *π* phase shift in the quantum oscillation has broader origins than the *π* Berry phase of a Dirac fermion because of the orbital magnetic moment and Zeeman effect. The success of manifesting the Berry phase of graphene lies in the negligible spin-orbital coupling (SOC)^42,43^, resulting in a zero orbital magnetic moment-induced phase *ϕ*_*R*_ and a small Zeeman effect-induced phase *ϕ*_*Z*_ as a spin reduction factor in the quantum oscillation intensity. In spinful systems (the SOC of *A*V_3_Sb_5_ is about dozens of meV), the non-zero *ϕ*_*R*_ and *ϕ*_*Z*_, due to spin-momentum locking sensitive to the Fermi energy, cannot be separated from *ϕ*_*B*_ in the total phase shift. As a result, the SOC-driven band anti-crossing can generate quantum orbits with a *π* phase shift in a proper energy window. Thus, the origin of observed nontrivial Fermi pockets of CsV_3_Sb_5_ may deviate from the vaguely argued *π*-Berry phase mechanism, which remains an open question. Furthermore, the dissimilar frequencies of nontrivial Fermi pockets of CsV_3_Sb_5_ remain to be reconciled.

Besides the discrepancy in the topological nature of Fermi pockets, another intriguing puzzle is their structural origin. Previous calculations based on the 2 × 2 × 1 CDW model of CsV_3_Sb_5_ cannot rationalize their respective quantum oscillation experiments^29,30^. The 2 × 2 × 1 CDW, i.e., the star of David (SD) and inverse star of David (ISD)^4^ structures, have been commonly adopted to understand various experiments and conceive theoretical models. Recent experiments suggest two-fold or four-fold interlayer modulation with alternative SD and ISD layers^6,29,44–48^. The interlayer structural modulation-induced FS reconstruction, which may help to understand the emergence of novel density wave orders and superconductivity at lower temperatures^49^, remains elusive.

In this work, we resolved the origin and topology of the observed quantum orbits in CsV_3_Sb_5_ from theory. We derived Fermi-energy-resolved and layer-resolved quantum orbits that agree quantitatively with experiments in the cyclotron frequency and nontrivial phase shift. According to the layer-contribution, we classified all quantum orbits into three groups, i.e., SD, ISD, and mixed (SD + ISD) group, where most small quantum orbits show a clear 3D nature. According to the topological origin, the nontrivial quantum orbits are classified into four types, with the topology of one type originating from the *π* Berry phase and the other three originating from the SOC jointly with the Zeeman effect. Most importantly, the SOC and/or Zeeman effect can lead to a trivial quantum orbit (zero phase shift in the quantum oscillation), even though the orbit has a *π* Berry phase in the spinless case. A hidden Dirac nodal network protected by mirror symmetries in the 3D CDW phase is revealed, which is weakly gapped in the presence of SOC^50,51^. We not only resolve the topology of quantum orbits but also reveal the appreciable 3D nature of Fermi surfaces in CsV_3_Sb_5_.

## Results and discussion

### Fermi surface of CsV_3_Sb_6_

**Fig. 1:**
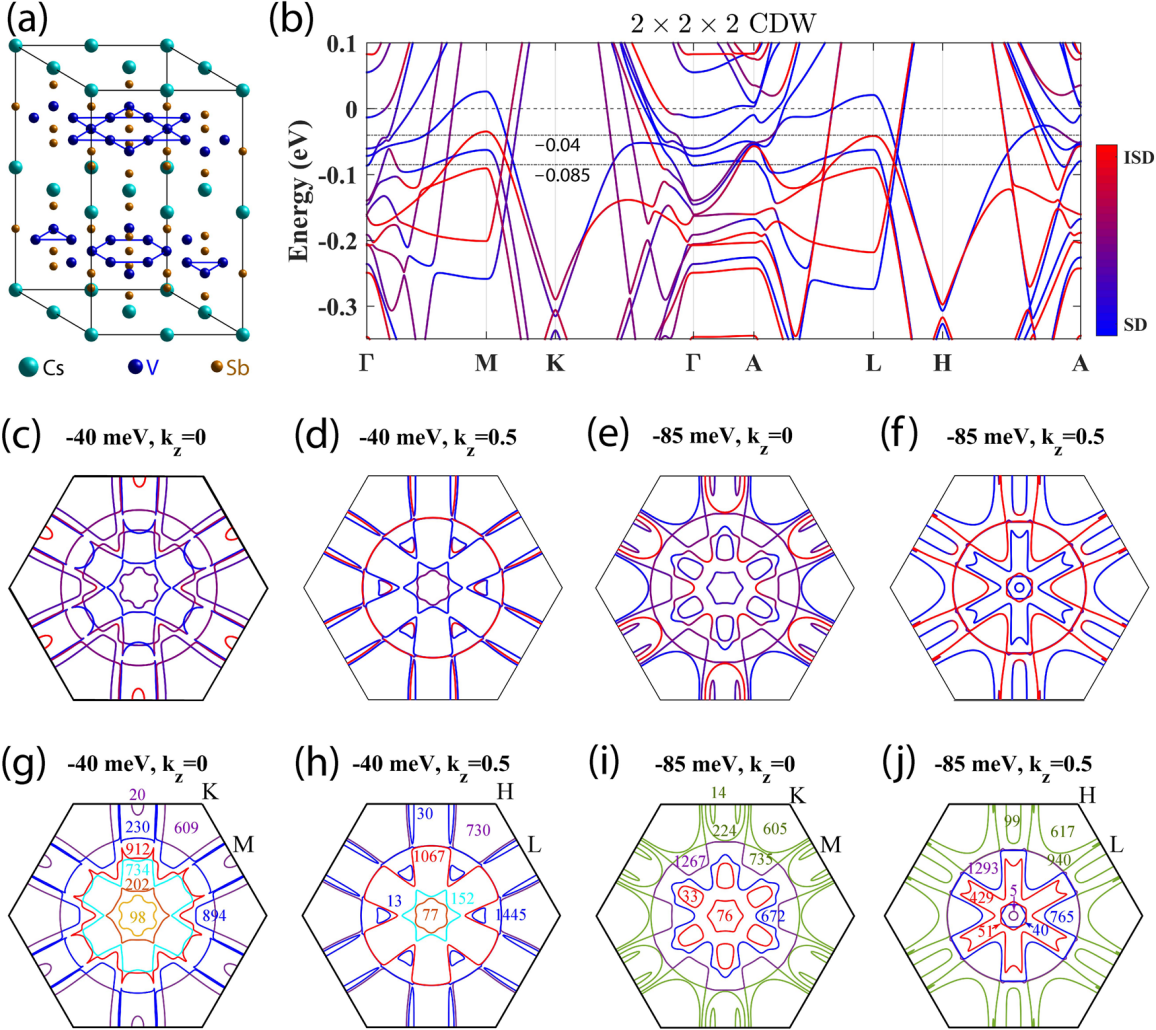
Crystal structure and layer-resolved band structure and Fermi surfaces of the 2 × 2 × 2 CDW. **a** The crystal structure with alternating SD and ISD layers. **b** Band structure. The color bar indicates the ISD (red) and SD (blue) contributions. The energy of the charge neutral point is set to zero. Energies of −40 and −85 meV are indicated by dash-dotted lines. **c**–**f** Layer-resolved Fermi surfaces in the first Brillouin zone (BZ) of the CDW state at −40 and −85 meV on *k*_*z*_ = 0 and 0.5 (2*π*/*c*) planes. The color bar is the same as (**b**). **g**–**j** show cyclotron frequencies (in units of T) of the corresponding Fermi surfaces where the line color differentiates different Fermi surfaces. These seemingly open Fermi surfaces at the BZ boundary extend to neighboring BZs and form closed Fermi pockets. SOC is included in calculations.

We consider three hexagonal CDW structures with the *P*6/*mmm* space group symmetry, i.e., the 2 × 2 × 1 SD and ISD and the 2 × 2 × 2 CDW. The energetic stability of interlayer stacking has been theoretically studied^4,7,9,29,52^. We adopt the 2 × 2 × 2 CDW alternating SD and ISD layers without interlayer lateral shift as suggested by recent APRES measurements^47,48^, shown in Fig. 1a. As we will see, the 2 × 2 × 2 model captures the essential interlayer interaction, which also occurs in more complicated structures like the 2 × 2 × 4 CDW^29^.

The band structure calculated by density-functional theory (DFT) is shown for the 2 × 2 × 2 CDW in Fig. 1b. By layer resolution, bands and FSs are well separated into SD, ISD, and mixed SD + ISD types. The band structure can be regarded as overlapping SD and ISD phases with considerable hybridization (see Supplementary Fig. 1). Because the chemical potential in experiments^11,23,24,29,53^ is usually lower than the theoretical charge neutral point (energy zero), we will focus on the Fermi energy window of −100 to 0 meV and investigate corresponding FS properties. We show two typical Fermi energies at –40 and –85 meV in Fig. 1c–j to demonstrate the layer- and energy-dependence of multiple quantum orbits.

**Fig. 2:**
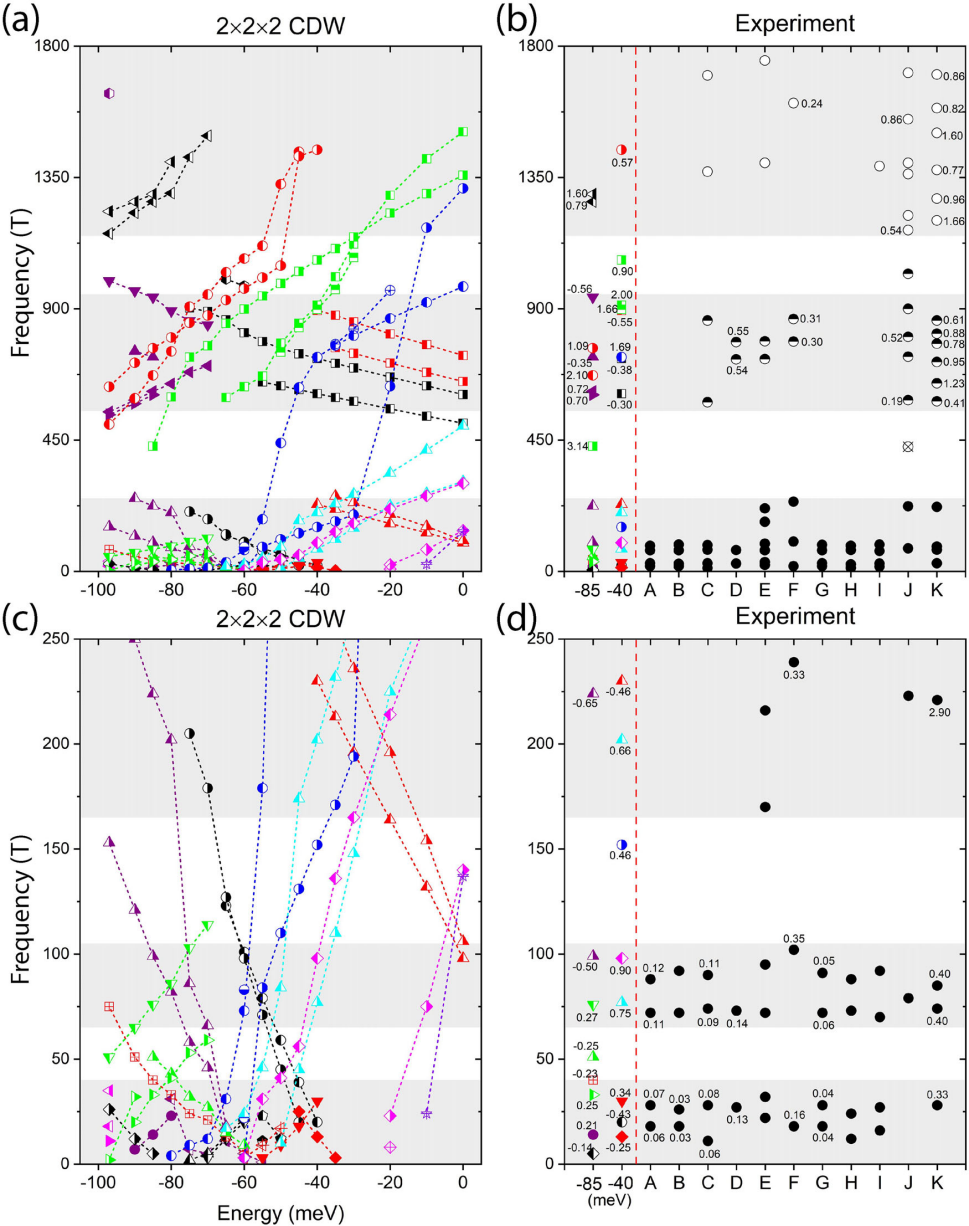
Calculated cyclotron frequencies in comparison with experiments. **a** Cyclotron frequencies of extremal quantum orbits as a function of Fermi energy for the 2 × 2 × 2 CDW. The energy of the charge neutral point is set to energy zero. Symbols with the same color and style connected by dashed lines represent the evolution of the same quantum orbit. **b** Comparison of calculated cyclotron frequencies at −40 and −85 meV (on the left side of the red dashed line) with experimental values in literature. Experimental set A is from ref. ^31^, B from ref. ^28^, C from ref. ^29^, D from ref. ^30^, E and F from ref. ^32^ for the angle between the magnetic field and *c* axis being 0^∘^ and 20^∘^, respectively, G from ref. ^33^, H and I from ref. ^34^ for bulk and flake, respectively, J from ref. ^35^, and K from ref. ^36^. Numbers marked near the symbols are corresponding cyclotron masses in units of *m*_0_. The experimental values are divided into three regions, i.e., low-frequency region 0–250 T, medium-frequency region 550–950 T, and high-frequency region ≥1150 T. **c**, **d** are a zoom-in of the low-frequency region in (**a**) and (**b**), respectively, which can be further divided into three sub-regions as indicated by the three gray backgrounds.

We obtain extremal quantum orbits by extracting the maximal and minimal FS area along the *k*_*z*_ axis for the 2 × 2 × 2 CDW and show their Fermi energy dependence in Fig. 2a, c. Most extremal orbits distribute in high-symmetry planes of *k*_*z*_ = 0/0.5 (in a unit of 2*π*/*c*, *c* is the out-of-plane lattice parameter), while some special cases appear at a generic *k*_*z*_ (see an example at –40 meV in Table 1). We will identify the proper Fermi energy by comparing calculated cyclotron frequencies with experimental values.

**Table 1:** Quantum orbits of the 2 × 2 × 2 CDW at −40 and −85 meV.

−40 meV								−85 meV							
Freq.	*k*_*z*_	m^*^	Origin	*ϕ*_*B*_	*λ*	Δ*ϕ*	Type	Freq.	*k*_*z*_	m^*^	origin	*ϕ*_*B*_	*λ*	Δ*ϕ*	Type
(T)	(2*π*/*c*)	(*m*_0_)		(*π*)	(*π*)	(*π*)		(T)	(2*π*/*c*)	(*m*_0_)		(*π*)	(*π*)	(*π*)	
13	0.5	−0.25	mixed	1	0.68	1	I	5	0.5	−0.14	SD	0	0.93	1	II
20	0	−0.43	ISD	0	0.38	0		14	0	0.21	mixed	1	0.77	1	I
30	0.5	0.34	mixed	1	0.83	1	I	33	0	0.25	SD	0	0.84	1	II
77	0.5	0.75	mixed	0	0.92	1	III	40	0.5	−0.23	SD	0	0.44	0	
98	0	0.90	mixed	0	0.87	1	IV	51	0.5	−0.25	ISD	0	0.52	1	IV
152	0.5	0.46	SD	0	0.98	1	II	76	0	0.27	SD	0	0.02	0	
202	0	0.66	SD	0	0.37	0		99	0.5	−0.50	mixed	0	0.89	1	II
230	0	−0.46	mixed	0	0.84	1	II	224	0	−0.65	ISD	0	0.57	1	III
609	0	−0.30	mixed	0	0.48	0		429	0.5	3.14	SD	0	0.06	0	
730	0.5	−0.38	SD	0	0.92	1	II	605	0	0.70	SD	1	0.38	0	
734	0	1.69	SD	0	0.22	0		617	0.5	0.72	SD	1	0.35	0	
894	0	−0.55	mixed	0	0.19	0		672	0	2.10	mixed	0	0.11	0	
898	0.1	1.66	mixed	0	0.71	1	IV	735	0	−0.35	mixed	0	0.55	1	IV
912	0	2.00	mixed	0	0.31	0		765	0.5	1.09	ISD	0	0.60	1	III
1067	0.5	0.90	SD	0	0.33	0		940	0.5	−0.56	ISD	0	0.07	0	
1445	0.5	0.57	ISD	0	0.26	0		1267	0	0.79	mixed	0	0.25	0	
								1293	0.5	1.60	mixed	0	0.08	0	

Frequency (Freq.) is in unit of T. kz refers to the kz plane (in a unit of 2π/c, c is the lattice constant) the corresponding quantum orbit located in. The wavy-underlined kz indicates a minimal Fermi surface cross-section and the others correspond to maximal ones. The maximal/minimal orbit provides helpful information to determine the sign of the extra π/4 phase shift when analyzing quantum oscillations of a three-dimensional material. The cyclotron mass m* is in a unit of bare electron mass m0, where positive and negative values are for electron and hole pockets, respectively. The origin of quantum orbits (i.e., layer-contribution) is indicated by SD, ISD, and mixed (SD+ISD), as obtained from Fig. 1(b)–(f). The Berry phase ϕB of the quantum orbit is calculated without SOC and the generalized Berry phase λ is calculated with SOC (reduced to the range of –π to π). All quantum orbits are doubly degenerate and have λ of the same magnitude but reversed signs (only the positive set is listed). Depending on ∣λ∣ being either smaller or larger than 0.5π, the final phase shift (Δϕ) in quantum oscillation contributed by two degenerate quantum orbits is either 0 or π. The four types of nontrivial quantum orbits are defined in the main text and classified according to Table S1 and S2 in Supplementary.

We summarized exhaustively recent quantum oscillation measurements^28–36^ in Fig. 2b, d. Most experimental results can be divided into three groups, i.e., the low-frequency region 0–250 T, medium-frequency region 550–950 T, and high-frequency region ≥1150 T. Many high frequencies may be induced by the magnetic breakdown by merging neighboring FSs^41^. Thus, we will focus on cyclotron frequencies no larger than 1800 T. There are 4–6 cyclotron frequencies in the low-frequency region, 2–6 frequencies in the medium-frequency region, and 1–6 frequencies in the frequency region of 1150–1800 T.

Considering both the number and distribution of frequencies in three regions, we find theoretical results at energy ranges of [−50, −35] meV and [−90, −75] meV match well with most experiments. One may also find results in the range of [−20, 0] meV match experiments at medium- and high-frequency regions. However, the agreement in the low-frequency region in this energy range is poor [see Fig. 2c, d]. In the following, we will analyze two representative Fermi energies, −40 and −85 meV, as shown in Fig. 1 and Table 1. In detail, the 2 × 2 × 2 CDW shows 8 frequencies in the low-frequency region and 7 frequencies in the medium-frequency region at both −40 and −85 meV. In the high-frequency region, it shows 1 frequency at −40 meV and 2 frequencies at −85 meV. Due to band folding and hybridization in the 2 × 2 × 2 CDW, the large Fermi surfaces in the pristine phase break into relatively smaller pieces. Thus theoretical results in low- and medium-frequency regions generally agree better with experiments than the high-frequency region, as shown in Fig. 2. The highest frequencies at −40 or −85 meV correspond to nearly isotropic FSs centered at Γ (see Fig. 1), which originates in Sb-*p*_*z*_ bands. Corresponding FSs are very close to several neighboring pockets. Thus, magnetic breakdown may occur to generate even larger frequencies in experiments, rationalizing the magnetic breakdown observed in ref. ^35^.

In contrast, we cannot find a satisfactory agreement between experiments and the 2 × 2 × 1 CDW (SD or ISD) simultaneously in all three frequency regions. The quantitative comparison similar to Fig. 2 is shown in Supplementary Fig. 2. Our results are consistent with refs. ^29,30^, where the SD or ISD could not match all frequencies in their respective experiments.

Furthermore, we compare with experiments the cyclotron mass m^*^ (in a unit of bare electron mass *m*_0_) of quantum orbits in Table 1 and Fig. 2. In the low-frequency region in Fig. 2d, reported masses are rather diverse in literature. Several experiments found m^*^ = 0.03–0.14, while ref. ^32^ and ref. ^36^ found m^*^ = 0.16–0.40, [sets F and K in Fig. 2d]. Our calculations agree better with ref. ^32^ and ref. ^36^ (except the large value of 2.90 for the observed frequency 221 T) in the low-frequency region. In the medium- and high-frequency regions, experiments^30,32,35,36^ found effective masses typical from 0.3 to 1.7. In general, our calculated m^*^ at −40 and −85 meV are within experimental ranges, by considering the uncertainty of Fermi energy in experiments. Therefore, we conclude that our calculations considering the interlayer structural modulation agree with recent quantum oscillation experiments in both fundamental frequency and cyclotron mass.

These quantum orbits exhibit interesting interlayer hybridization, as shown in Fig. 1c–f and Table 1. Approximately half of the FSs at –40 and –85 meV are solely contributed by either SD or ISD layer, and the rest show a strongly mixed character of two layers, labeled as mixed in Table 1. There are two ways to form a mixed SD + ISD FS. In one way, part of the Fermi contour is contributed by the SD layer and the rest by ISD with weak hybridization between two layers. This happens mostly in the *k*_*z*_ = 0.5 plane, for example, for orbits of 13 and 30 T at −40 meV. The other way involves strong hybridization between two layers and the Fermi contour has comparable contributions from both layers almost everywhere. This happens mostly in the *k*_*z*_ = 0 plane, which is the case for most mixed quantum orbits at *k*_*z*_ = 0. Therefore, the interlayer structural modulation is crucial in determining the nature of FSs for the CsV_3_Sb_5_ CDW phase. Additionally, one can find by comparing the *k*_*z*_ = 0 and 0.5 planes that many large FS pockets are quasi-2D while many small FSs are 3D in geometry.

### Topological properties of quantum orbits

Next, we discuss the topological properties of these quantum orbits that are debated in experiments. In the following, the sum of the Berry phase, orbital and Zeeman phases under SOC is dubbed the generalized Berry phase *λ*, i.e., *λ* = *ϕ*_*B*_ + *ϕ*_*R*_ + *ϕ*_*Z*_. According to previous discussions^40^, symmetries of quantum orbits introduce additional constraints on *λ*. For CsV_3_Sb_5_ with both inversion and time-reversal symmetries, all bands are doubly degenerate with opposite generalized Berry phases ± *λ* (reduced to –*π–π*) in the spinful case. The superposition of the doublets leads to a $2| \cos (\lambda )|$ scaling of the quantum oscillation intensity, and the *λ* itself is no longer the total phase shift of the quantum oscillation. Instead, the quantum oscillation always exhibits a quantized phase shift Δ*ϕ* (see the method section and Supplementary Notes 1–3). Here, Δ*ϕ* is 0 or *π*, depending on whether the reduced ∣*λ*∣ is smaller/larger than 0.5*π*. According to the origin of *λ*, nontrivial quantum orbits characterized by Δ*ϕ* = *π* (or ∣*λ*∣ > 0.5*π*) in CsV_3_Sb_5_ can be classified into four types. Type-I refers to the Dirac point-driven quantum orbit exhibiting a robust *ϕ*_*B*_ = *π* and small *ϕ*_*R*_ and *ϕ*_*Z*_. Type-II shows strong SOC-induced large *ϕ*_*B*_+*ϕ*_*R*_ with a small *ϕ*_*Z*_. In type III, the *ϕ*_*Z*_ is dominant over the *ϕ*_*B*_+*ϕ*_*R*_. In type IV, both the SOC-induced *ϕ*_*B*_+*ϕ*_*R*_ and *ϕ*_*Z*_ are crucial. We point out that strong SOC and/or Zeeman effect can also suppress the *λ* heavily (∣*λ*∣ < 0.5*π*) for some quantum orbits with a robust *ϕ*_*B*_ of *π*.

**Fig. 3:**
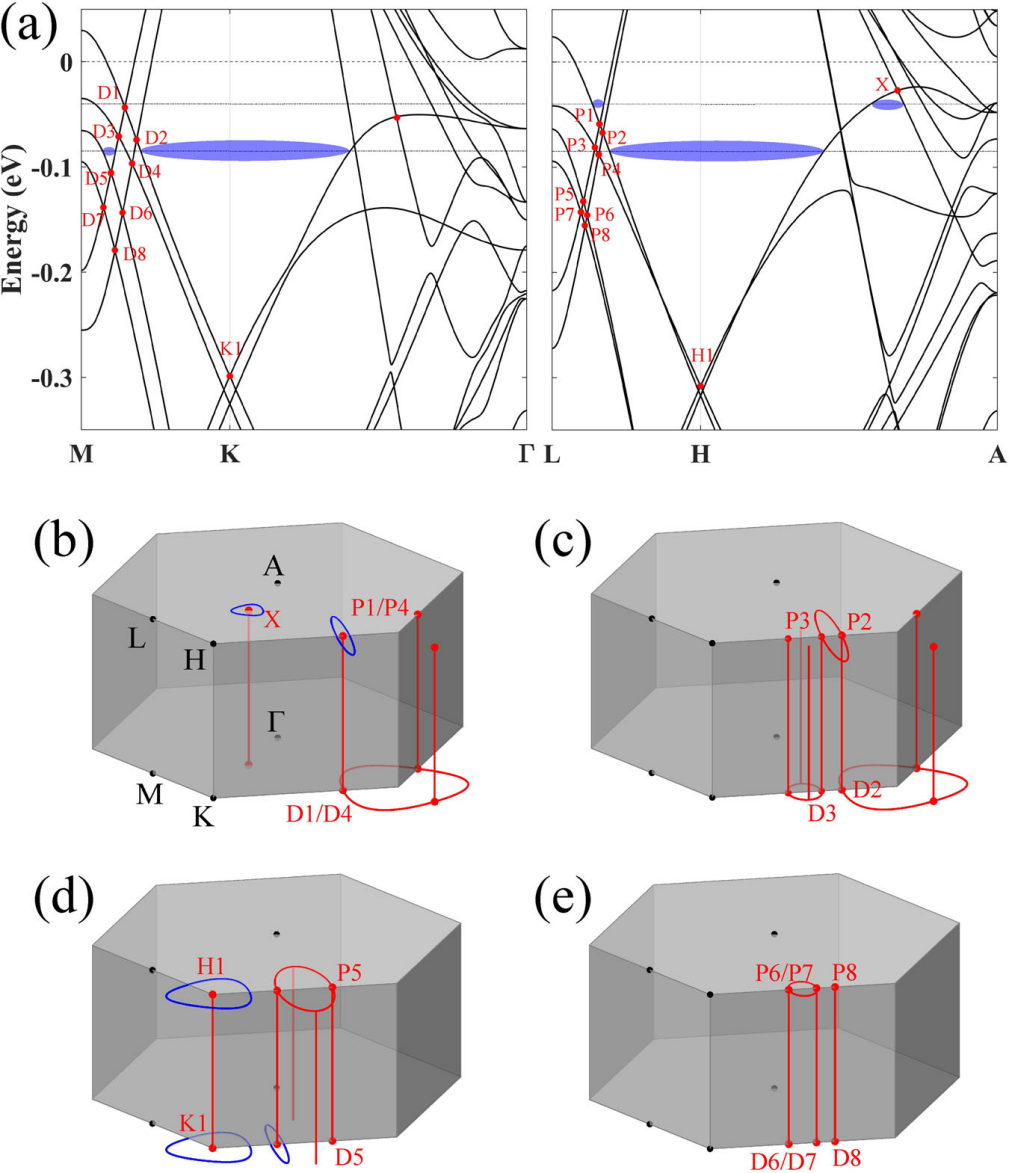
Dirac nodal lines and nodal networks in the band structure. **a** Band structures on *k*_*z*_ = 0 (left part) and 0.5 (right part) planes without SOC. Eight Dirac points on the *MK* (*LH*) line near the charge neutral point are labeled from D1 to D8 (P1–P8), respectively. Selected Dirac points at *K* and *H* are labeled as *K*1 and *H*1, respectively. The two dash-dotted horizontal lines indicate the energy positions of −40 and −85 meV, respectively, where nontrivial Fermi surfaces (*ϕ*_*B*_ = *π*) are indicated by blue ellipses. Dirac nodal lines/rings connecting D1 to D8 (P1–P8) Dirac points are divided into four groups, as sketched in (**b**–**e**). Blue circles in (**b**) and (**d**) indicate Fermi surfaces surrounding a nodal line at −40 and −85 meV in (**a**, **b**), respectively. For simplicity, we show those Dirac nodal lines/rings and Fermi surfaces partly near one *M*/*L* and one *K*/*H* point. The full Dirac nodal lines/rings and nontrivial Fermi surfaces can be obtained by crystal symmetries (*P*6/*mmm*). We note that nodal lines except the *K*1 − *H*1 line are not necessarily straight along *k*_*z*_ but locate inside the mirror plane.

We first calculate the accumulated Berry phase along the quantum orbit in the 2 × 2 × 2 CDW phase using the Wilson loop method^54–56^ without including SOC. To demonstrate the type-I quantum orbits, we show the band structure without SOC in Fig. 3(a). At the *K*/*H* point, one can find Dirac points in *k*_*z*_ = 0/0.5 planes caused by the kagome structure symmetry, where we mark the higher Dirac point as *K*1/*H*1. However, these Dirac points cannot be isolated band crossing points because they locate inside mirror planes in the 3D momentum space. Otherwise, they would exhibit a monopole-like Berry charge which violates the mirror symmetry. Consequently, Dirac points expand inside the mirror plane and present a continual nodal line/ring. Along the *K* − *H* line, there indeed exists a Dirac nodal line connecting *K*1 and *H*1. As shown in Fig. 3d, quantum orbits surrounding the nodal line show a *π* Berry phase, which is the case of two medium-frequency orbits (605 and 617 T) at the Fermi energy –85 meV (Table 1). After considering SOC, however, the generalized Berry phases *λ* of these two quantum orbits are heavily reduced (∣*λ*∣ < 0.5*π*) by the large Zeeman phases (roughly proportional to the cyclotron mass, see Supplementary Table 2). Thus such orbits are trivial in quantum oscillation showing no phase shifts. Recent experiments indeed observed quantum orbits with similar frequencies (580–590 T^29,35,36^ and 645 T^36^). However, none of them shows a *π* phase shift. Furthermore, there are additional Dirac points due to band crossings on the *M* − *K* line [D*i*, *i* = 1–8, see Fig. 3a] and also on the *L* − *H* line (P*i*). Because of the mirror symmetry with respect to the *MKH* plane, D*i* and P*i* are linked by a nodal line inside the *MKH* plane and related bands have opposite mirror parities. Because *k*_*z*_ = 0 and 0.5 planes are mirror planes, some of D*i*/P*i* extend further inside the *k*_*z*_ = 0/0.5 planes and form nodal rings. Therefore, intriguing Dirac nodal networks with linked nodal lines and nodal rings emerge in the momentum space. For example, D1 & P1 (also P4 & D4 and D5/6/7 & P5/6/7) are connected to form a cylinder-like network, and P2 & P3 are linked to constitute a 3D network. For the nodal network, the quantum orbit can exhibit a *π* Berry phase if it surrounds one nodal line and avoids crossing other nodal lines/rings. This is the case for the D5-induced FS (14 T at –85 meV) in Fig. 3d and P1-induced FS (30 T at –40 meV) in Fig. 3b which are type-I quantum orbits (Table 1). In contrast, a Dirac-like crossing cannot generate a nontrivial FS in a plane if there is also a related nodal ring in the same plane. For example, D1/D4 fails to create a nontrivial FS in the *k*_*z*_ = 0 plane. In addition, there is another nodal line crossing the *X* point [Fig. 3(b)], leading to a *π* Berry phase of the 13 T orbit at –40 meV, which belongs to the type-I quantum orbits. These Dirac nodal lines and networks may generate nontrivial quantum orbits in a relatively large energy range besides –40 and –85 meV. We note that Dirac nodal lines were reported in the pristine phase of *A*V_3_Sb_5_^2,57,58^ rather than the CDW state.

Other three types of nontrivial quantum orbits originated from either strong SOC and/or strong Zeeman effect, are shown in Table 1 (see details in Supplementary Tables 1, 2). Most type II to type IV nontrivial quantum orbits are centered at time-reversal invariant points [i.e., Γ, *A*, *M*, and *L* points, see Fig. 3b]. Thus their Berry phases *ϕ*_*B*_ and orbital phases *ϕ*_*R*_ are strictly zero without SOC due to the time-reversal symmetry constraint (see Supplementary Note 2). Only when SOC is involved the *ϕ*_*B*_ and *ϕ*_*R*_ can appear in quantum orbits.

Overall, we found sixteen nontrivial quantum orbits at two Fermi energies. More than half of them originate from a mixture of SD and ISD layers. We list calculated nontrivial quantum orbits and the experimental ones in Table 2 for convenience of comparison. Except for extremely high-frequency orbits, our calculations agree well with experiments in the fundamental frequency, cyclotron mass, and topology.

**Table 2:** Frequencies of calculated and experimentally observed nontrivial quantum orbits.

Exp.	−40 meV	−85 meV
		5
18^32^	13	14
28^36^	30	33
73^30^, 74^36^, 79^35^, 85^36^	77	51
102^32^	98	99
	152	
239^32^	230	224
727^30^, 736^35^, 788^32^, 804^35^	730	735, 765
865^32^	898	
1605^32^		
2135^32^		

Recently, Zhou and Wang proposed an effective single-orbital model for the 2 × 2 × 1 CDW with time-reversal symmetry breaking loop-currents^49^, where reconstructed small Chern Fermi pockets carrying concentrated Berry curvature of the Chern band emerge at *M* points. These pockets are connected by one-quarter and three-quarters of the primitive reciprocal lattice vectors and may play a crucial role in the observed pair density wave order in the superconducting state^5^. We verified the existence of such Fermi pockets at *M* for the realistic 2 × 2 × 2 CDW. They can be seen at –40 (20 T) and –85 meV (99 T) in Fig. 1g, j, respectively. Moreover, we find that under SOC, the 20 T pocket does not have a Berry phase without time-reversal symmetry breaking, while the 99 T pocket has a Berry phase close to *π*. Upon closer examination (Supplementary Fig. 4 for FS unfolding), the 20 T pocket around *M* at –40 meV originates from the ISD layer due to folding a pocket centered at the crossing point between Γ − *K* and *M* − *M* in the primitive Brillouin zone, which can be captured by a 2 × 2 × 1 real CDW with ISD bond order^59^. In contrast, the 99 T pocket in the *k*_*z*_ = 0.5 plane is of mixed ISD and SD character generated by folding a pocket at the *k*_*z*_ = 0.25 plane in the primitive zone, which is unique to the alternate stacking of the ISD and SD layers of the 3D CDW.

Additionally, recent Raman experiments^60,61^ and time-resolved angle-resolved photoemission spectroscopy^62^ detected CDW-driven phonon modes. Measured CDW modes can be described by intralayer vibrations, except that the lowest-energy phonon is attributed to the interlayer Cs vibrations. Compared to phonons, electrons experience a more three-dimensional CDW structure and show stronger interlayer interaction in CsV_3_Sb_5_. The present 2 × 2 × 2 CDW is a minimal model to capture the interlayer coupling and reproduce all quantum orbits measured in experiments (except large orbits due to magnetic breakdown). If we adopt the 2 × 2 × 4 models in calculations, it will bring a much larger number of quantum orbits and complicate the physics understanding.

We point out that Dirac nodal lines and networks may be a general characteristic of kagome materials. For example, the *K*1 − *H*1 nodal line comes from the characteristic Dirac points of the Kagome lattice. This scenario applies both to nonmagnetic and magnetic kagome materials. For instance, a similar *K*1 − *H*1 nodal line was also found in the noncollinear kagome antiferromagnets Mn_3_Sn/Ge^63^. Because many mirror planes (e.g., the *MKH* plane and Γ*ML*) exist, degenerate band crossing points form nodal lines inside mirror planes and nodal lines from intersecting planes may link to each other. In the presence of strong SOC, these nodal lines are usually gapped out^50,51^ and generate large Berry curvature^64^ or spin Berry curvature^65^. Some nodal lines also lead to drumhead-like topological surface states^66^ on a specific facet.

In summary, we studied the Fermi surface properties of the 3D CDW in CsV_3_Sb_5_. The comprehensive analysis of quantum orbits indicates that the 2 × 2 × 2 CDW with interlayer structural modulation agrees broadly with recent quantum oscillation experiments in fundamental frequencies, cyclotron mass, and nontrivial phase shifts. The nontrivial Berry phases reveal a hidden Dirac nodal network in the momentum space due to mirror symmetry protection in the spinless case. We advanced the study of quantum oscillations in layered materials by identifying the different structural and topological origins of all quantum orbits. Our work not only resolves the Fermi surface puzzle in CsV_3_Sb_5_ but also serves as the first realistic example to show the crucial role of SOC in determining the topology of quantum orbits in quantum oscillation experiments.

## Methods

### Fermi surface calculation

We have performed density-functional theory (DFT) calculations with the Vienna ab initio Simulation Package (VASP)^67,68^ and fully relaxed all structures. Except for structural relaxation, spin-orbital coupling (SOC) is considered in electronic structure calculations if otherwise stated. More details about DFT calculations are referred to in ref. ^4^. High-resolution Fermi surfaces are calculated via Wannier functions^69^ (V-*d* and Sb-*p* orbitals) extracted from DFT. *k*-meshes of at least 100 × 100 × 100 and 100 × 100 × 50 are used to calculate band structures in the full BZs of 2 × 2 × 1 and 2 × 2 × 2 CDWs, respectively. The in-plane extremal (maximal and minimal) orbits of the closed FS at different energies, which corresponds to the magnetic field parallel to the *c* axis in quantum oscillation experiments, are determined by tracking the FS slice changes along the *k*_*z*_ direction. The cyclotron frequency of an electron on a closed quantum orbit is calculated according to Onsager’s relation^70^, $F=\frac{\hslash c}{2\pi e}{A}_{e}$, where *A*_*e*_ is the area of an extremal orbit at Fermi energy *ε*_*F*_, *e* is the electron charge and ℏ is the reduced Planck constant. The effective mass of a cyclotron electron is calculated with ${m}^{* }=\frac{{\hslash }^{2}}{2\pi {m}_{0}}\frac{\partial {A}_{e}}{\partial {\varepsilon }_{F}}$, where *m*_0_ is the bare electron mass.

### Quantum oscillation phase calculation

Comprehensive explanations of different phases in quantum oscillations are found in Supplementary Notes 1–3. Here we provide a brief introduction to how to calculate them. More theoretical backgrounds can be found in refs. ^40,41^.

In the quantum oscillation experiment, electrons undergo cyclotron motion whose phase interference condition gives rise to Landau level. There are mainly six contributions to the total phase and the quantization rule for the total phase is,

1 $${l}_{B}^{2}{A}_{e}+{\lambda }_{a}-{\phi }_{M}=2\pi n$$

where ${l}_{B}=\sqrt{\hslash c/eB}$ is the magnetic length. The first term is the sum of the de Broglie phase and Aharonov–Bohm (A-B) phase. *ϕ*_*M*_ is the Maslov correction and equals to *π* for a simple closed smooth curve (all the cyclotron orbits considered in this paper belong to this category). The generalized Berry phase *λ*_*a*_ (*a* = 1, 2 the degenerate band indices) has three contributions (Berry phase *ϕ*_*B*_, orbital phase *ϕ*_*R*_, and spin Zeeman phase *ϕ*_*Z*_) and can be calculated from the eigenvalue ${e}^{i{\lambda }_{a}}$ of the propagator U[*C*] of loop *C* ($\overline{\exp }$ means path-ordered exponential),

2 $$U[C]=\overline{\exp }\left\{i\oint \left[({{{\boldsymbol{A}}}}+{\mathfrak{A}})\cdot d{{{\boldsymbol{k}}}}+\frac{{g}_{0}\hslash }{4m{v}^{\perp }}{\sigma }^{z}| d{{{\boldsymbol{k}}}}| \right]\right\}$$

where ***A***_*mn*_ = *i*〈*u*_*mk*_∣***∇***_*k*_*u*_*nk*_〉 ($m,n\in {{\mathbb{Z}}}_{D}$, ${{\mathbb{Z}}}_{D}$ is the degenerate band group being considered.) is the non-Abelian Berry connection, and the first term is the Berry phase for the multiband case. The second phase, which comes from the orbital magnetic moment, is

3 $${{\mathfrak{A}}}_{mn}\cdot d{{{\boldsymbol{k}}}}=\mathop{\sum}\limits_{l\notin {{\mathbb{Z}}}_{D}}{A}_{ml}^{x}{\Pi }_{ln}^{y}d{k}_{x}/2{v}_{y}+(x\leftrightarrow y)$$

with **Π**_*ln*_ = 〈*u*_*lk*_∣***v***∣*u*_*nk*_〉 being the interband matrix elements of the group velocity operator ***v***. Here, we emphasize the importance of the orbital phase which is usually ignored in experiments. Since CsV_3_Sb_5_ has inversion and time-reversal symmetries, the orbital phase is identically zero for all quantum orbits without SOC. But in the presence of SOC, it may contribute non-negligibly to the total phase *λ*_*a*_. For example, it can compensate the loss of the *π* Berry phase in a Dirac model with a Semenoff mass^71^ (see also Supplementary Note 3). The last term comes from the spin Zeeman effect and is usually written as a reduction factor^72,73^ in quantum oscillation. When SOC is not included, the three terms can be calculated separately. Under SOC, the three contributions to the propagator must be calculated as a whole, so separating the spin reduction factor is no longer possible. In other words, the orbital and Zeeman phases must be calculated together with the Berry phase to get observable results.

The Berry phase, with or without the inclusion of the orbital phase and the generalized Berry phase *λ*_*a*_ calculated for quantum orbits of the 2 × 2 × 2 CDW are listed in Supplementary Table 1, S2. Due to the double degeneracy of quantum orbits ensured by both inversion and time-reversal symmetries, their generalized Berry phases are *λ*_1_ = + *λ* and *λ*_2_ = −*λ*. Thus the sum of two oscillations is

4 $$\begin{array}{lll}\cos ({l}_{B}^{2}{A}_{e}+{\lambda }_{1}-{\phi }_{M}\pm \frac{\pi }{4})+\cos ({l}_{B}^{2}{A}_{e}+{\lambda }_{2}-{\phi }_{M}\pm \frac{\pi }{4})\\ =2\cos (\frac{{\lambda }_{1}-{\lambda }_{2}}{2})\cdot \cos ({l}_{B}^{2}{A}_{e}+\frac{{\lambda }_{1}+{\lambda }_{2}}{2}-{\phi }_{M}\pm \frac{\pi }{4})\\ =2| \cos (\lambda )| \cdot \cos ({l}_{B}^{2}{A}_{e}+\Delta \phi -{\phi }_{M}\pm \frac{\pi }{4})\end{array}$$

where the final phase shift Δ*ϕ* is 0 or *π*, depending on whether $\cos (\lambda )$ is positive (∣*λ*∣ < 0.5*π*) or negative (∣*λ*∣ > 0.5*π*). If ∣*λ*∣ = 0.5*π*, the oscillation may not be observable because $\cos (\lambda )=0$. Notice that the $\pm \frac{\pi }{4}$ phase correction comes from the *k*_*z*_ integral in a three-dimensional case (it does not exist in a two-dimensional system).

### Supplementary information

Supplementary Material

## Data Availability

The data supporting the findings of this study are available within the paper and in the supplementary information.

## Code availability

All code used to calculate the presented results is available from the corresponding author upon reasonable request.
