# Supplementary material for: Emergent topological quantum orbits in the charge density wave phase of kagome metal CsV3Sb5
Source: NPJ Quantum Mater. 2023 Jul 28;8(1):39. doi: 10.1038/s41535-023-00571-w (PMC11041708; doi:10.1038/s41535-023-00571-w)
Supplement: Supplementary file 1 — Supplementary Material [file 41535_2023_571_MOESM1_ESM.pdf]

Supplementary Information for "Emergent topological quantum orbits in the charge density wave phase of kagome metal CsV_3Sb_5"

Hengxin Tan,[1] Yongkang Li,[1] Yizhou Liu,[1] Daniel Kaplan,[1] Ziqiang Wang,[2] and Binghai Yan[1]

[1]*Department of Condensed Matter Physics, Weizmann Institute of Science, Rehovot 7610001, Israel*
[2]*Department of Physics, Boston College, Chestnut Hill, Massachusetts 02467, USA*

Table of content for Supplementary figures, notes, and tables:

- Supplementary Figure 1 displays band structures of the 2×2×1 CDW structures SD and ISD.
- Supplementary Figure 2 shows cyclotron frequencies of extremal orbits of 2×2×1 SD and ISD structures.
- Supplementary Figure 3 displays the layer resolved band structure of the 2×2×2 CDW without spin-orbital coupling.
- Supplementary Figure 4 shows the unfolded Fermi surfaces of the 2×2×2 CDW at −40 meV and −85 meV.
- Supplementary Figure 5 shows all Fermi surfaces of the 2×2×2 CDW at different energies.
- Supplementary Note 1 provides a general derivation of the Lifshitz-Kosevich formula with discussions on the phase shift.
- Supplementary Note 2 discusses the Berry phase in a time reversal and inversion symmetric system.
- Supplementary Note 3 shows the π total phase of a Dirac Hamiltonian with Semenoff mass.
- Supplementary Table 1 and 2 show the different phases of the quantum orbits (with and without spin-orbital coupling) of the 2×2×2 CsV_3Sb_5 at –40 meV and –85 meV, respectively.

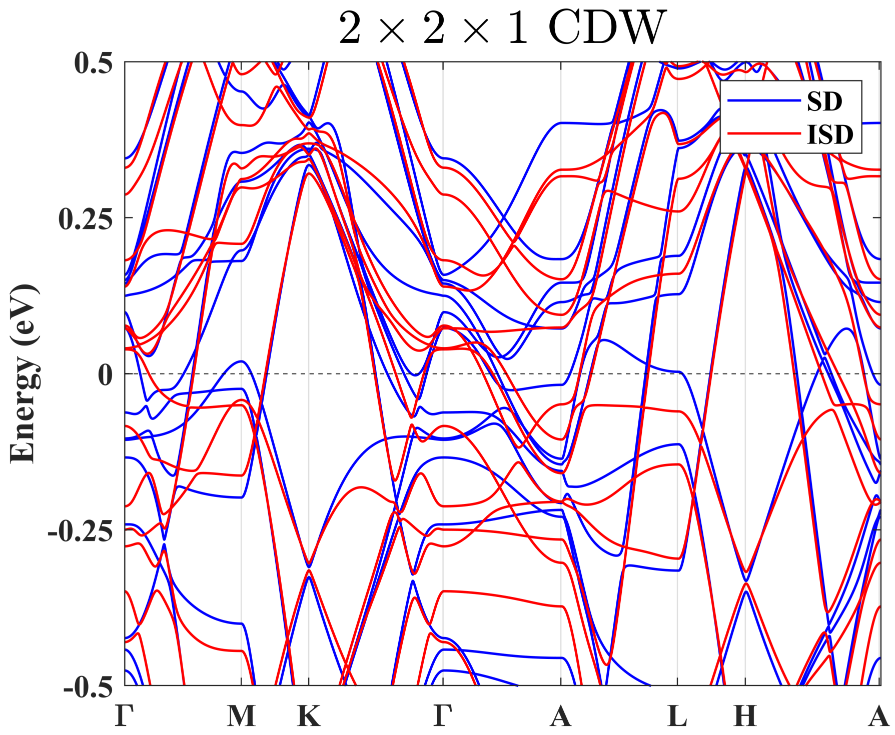

Supplementary Figure 1. Overlapping band structures of 2×2×1 CDWs of CsV_3Sb_5, blue for SD and red for ISD. Spin orbital coupling is considered. There is no coupling between SD and ISD.

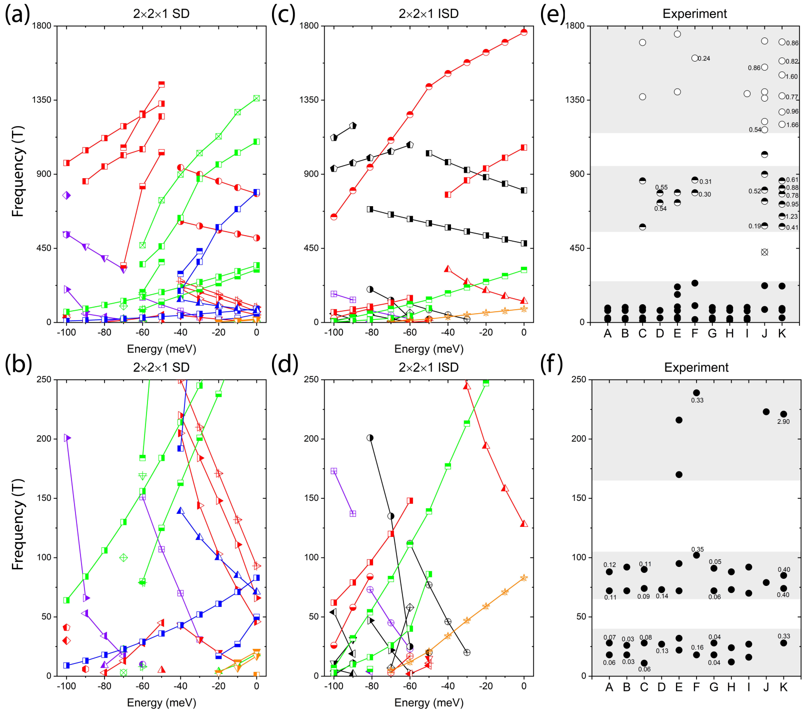

Supplementary Figure 2. Cyclotron frequencies of electrons on extremal orbits of 2×2×1 CDWs of CsV_3Sb_5. (a) and (b) are for SD. (c) and (d) are for ISD. (e) and (f) show the same experimental results as in the main text for comparison convenience. (a), (c) and (e) show the full range of 0∼1800 T while (b), (d), and (f) show the low-frequency region 0∼250 T.

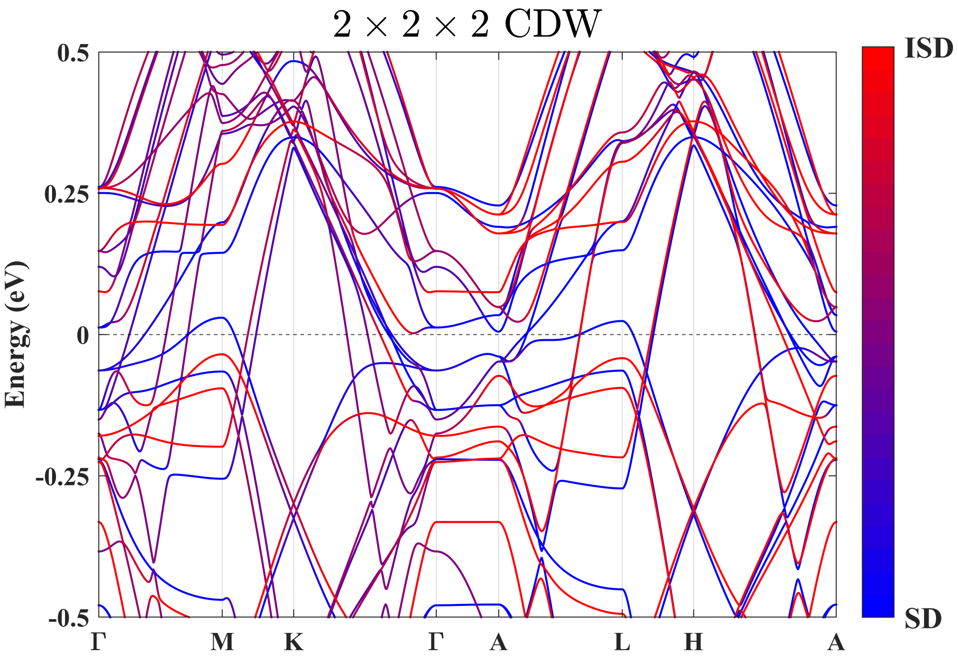

Supplementary Figure 3. Layer resolved band structure of the 2×2×2 CDW structure of CsV_3Sb_5 without considering spin-orbital coupling.

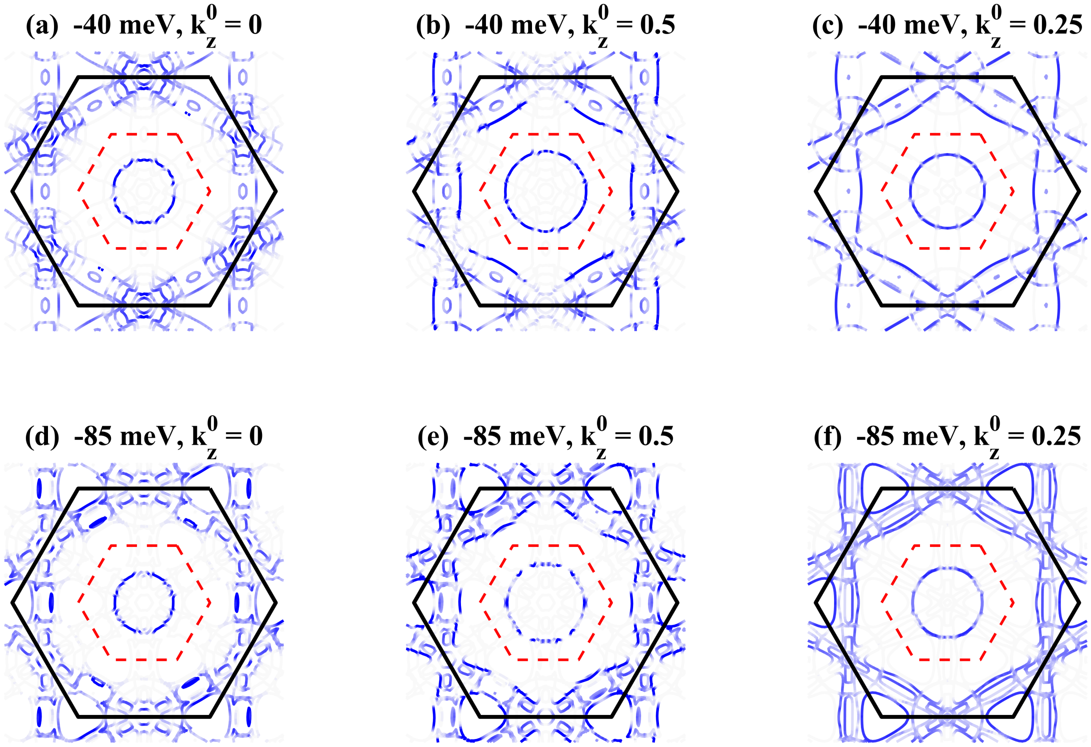

Supplementary Figure 4. Unfolded Fermi surfaces of the 2×2×2 CDW, (a-c) for −40 meV and (d-f) for −85 meV, respectively. The larger black solid hexagons show the Brillouin zone of the pristine phase (no CDW distortion), while the smaller red dashed hexagons show the Brillouin zone of the CDW structure. k_z and k_z^0 (in unit of $2\pi/c$) label planes of Brillouin zones of the CDW and pristine phases, respectively. Notice that the $k_z = 0$ plane of the 2×2×2 CDW phase is unfolded back to both $k_z^0 = 0$ and 0.5 planes of the pristine phase. The $k_z = 0.5$ plane of the 2×2×2 CDW phase is unfolded back to the $k_z^0 = 0.25$ plane of the pristine phase. The main features of these Fermi surfaces are similar, i.e., all show a large hexagonal Fermi surface near the Brillouin zone boundary and a circular Fermi surface around the Brillouin zone center.

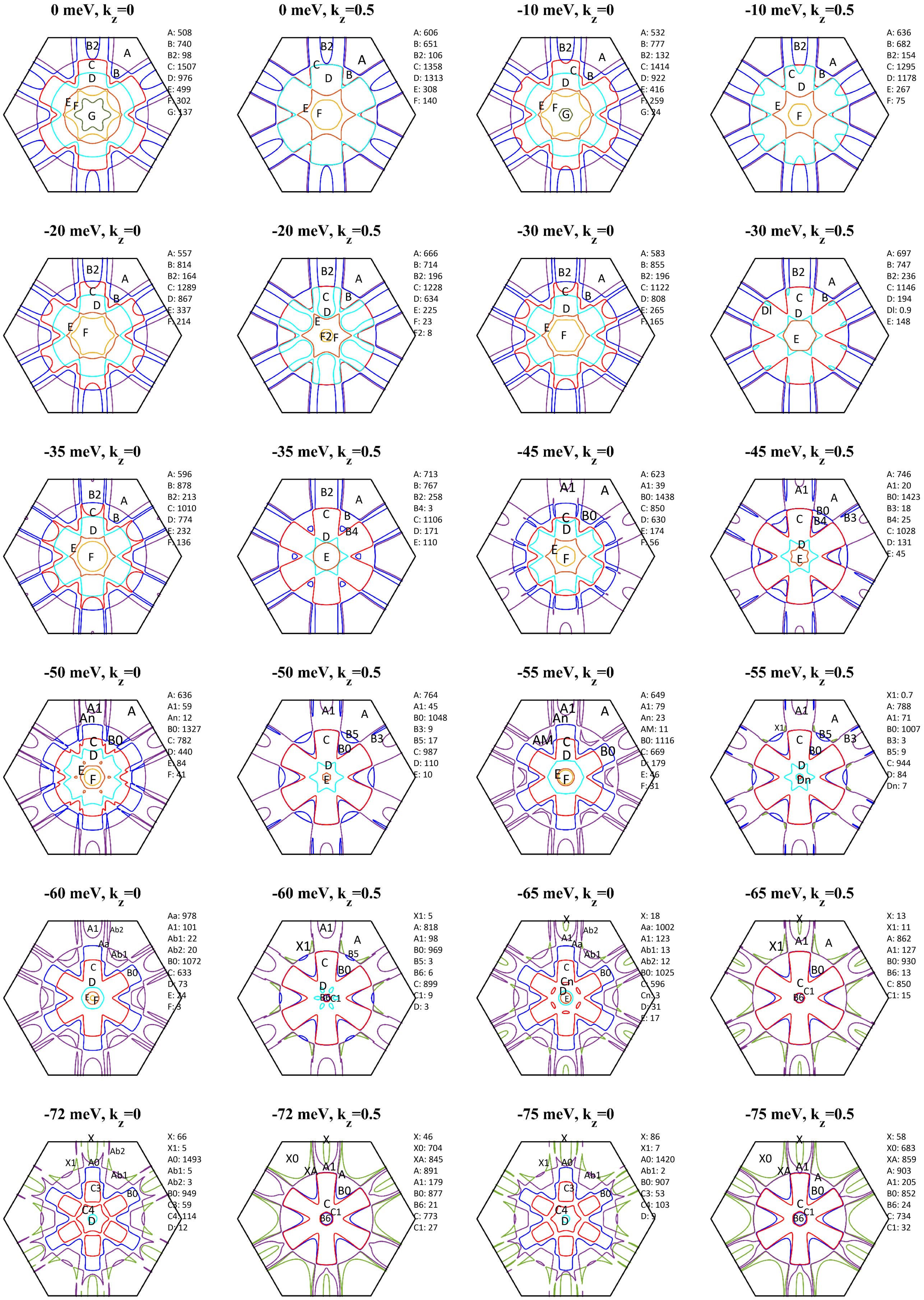
0 meV, k_z=0
A: 508
B: 740
B2: 98
C: 1507
D: 976
E: 499
F: 302
G: 137
0 meV, k_z=0.5
A: 606
B: 651
B2: 106
C: 1358
D: 1313
E: 308
F: 140
-10 meV, k_z=0
A: 532
B: 777
B2: 132
C: 1414
D: 922
E: 416
F: 259
G: 34
-10 meV, k_z=0.5
A: 636
B: 682
B2: 154
C: 1295
D: 1178
E: 267
F: 75
-20 meV, k_z=0
A: 557
B: 814
B2: 164
C: 1289
D: 867
E: 337
F: 214
-20 meV, k_z=0.5
A: 666
B: 714
B2: 196
C: 1228
D: 634
E: 225
F: 23
F2: 8
-30 meV, k_z=0
A: 583
B: 855
B2: 196
C: 1122
D: 808
E: 265
F: 165
-30 meV, k_z=0.5
A: 697
B: 747
B2: 236
C: 1146
D: 194
Dl: 0.9
E: 148
-35 meV, k_z=0
A: 596
B: 878
B2: 213
C: 1010
D: 774
E: 232
F: 136
-35 meV, k_z=0.5
A: 713
B: 767
B2: 258
B4: 3
C: 1106
D: 171
E: 110
-45 meV, k_z=0
A: 623
A1: 39
B0: 1438
C: 850
D: 630
E: 174
F: 56
-45 meV, k_z=0.5
A: 746
A1: 20
B0: 1423
B3: 18
B4: 25
C: 1028
D: 131
E: 45
-50 meV, k_z=0
A: 636
A1: 59
An: 12
B0: 1327
C: 782
D: 440
E: 84
F: 41
-50 meV, k_z=0.5
A: 764
A1: 45
B0: 1048
B3: 9
B5: 17
C: 987
D: 110
E: 10
-55 meV, k_z=0
A: 649
A1: 79
An: 23
AM: 11
B0: 1116
C: 669
D: 179
E: 46
F: 31
-55 meV, k_z=0.5
X1: 0.7
A: 788
A1: 71
B0: 1007
B3: 3
B5: 9
C: 944
D: 84
Dn: 7
-60 meV, k_z=0
Aa: 978
A1: 101
Ab1: 22
Ab2: 20
B0: 1072
C: 633
D: 73
E: 24
F: 3
-60 meV, k_z=0.5
X1: 5
A: 818
A1: 98
B0: 969
B5: 3
B6: 6
C: 899
C1: 9
D: 3
-65 meV, k_z=0
X: 18
Aa: 1002
A1: 123
Ab1: 13
Ab2: 12
B0: 1025
C: 596
Cn: 3
D: 31
E: 17
-65 meV, k_z=0.5
X: 13
X1: 11
A: 862
A1: 127
B0: 930
B6: 13
C: 850
C1: 15
-72 meV, k_z=0
X: 66
X1: 5
A0: 1493
Ab1: 5
Ab2: 3
B0: 949
C3: 59
C4: 114
D: 12
-72 meV, k_z=0.5
X: 46
X0: 704
XA: 845
A: 891
A1: 179
B0: 877
B6: 21
C: 773
C1: 27
-75 meV, k_z=0
X: 86
X1: 7
A0: 1420
Ab1: 2
B0: 907
C3: 53
C4: 103
D: 9
-75 meV, k_z=0.5
X: 58
X0: 683
XA: 859
A: 903
A1: 205
B0: 852
B6: 24
C: 734
C1: 32

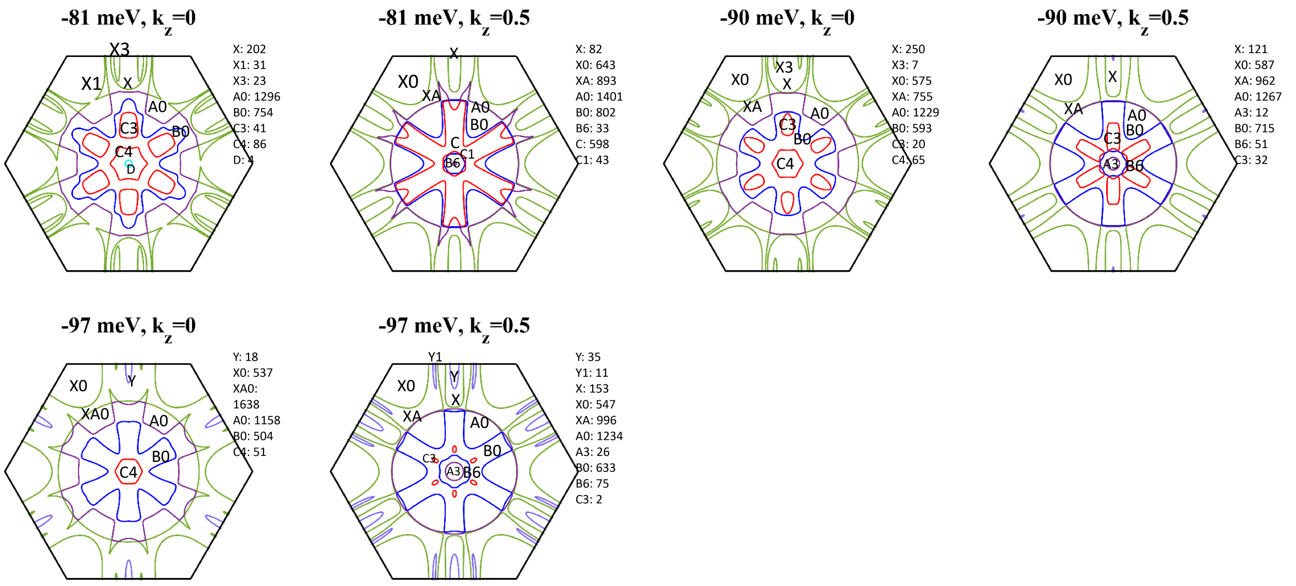

Supplementary Figure 5. Fermi surfaces (or constant energy contours) on $k_z = 0$ and 0.5 ($2\pi/c$) planes at different energies of the 2×2×2 CDW phase of CsV_3Sb_5 (SOC is considered). Corresponding cyclotron frequencies are given beside each panel (unit: T). Results at −40 and −85 meV are shown in the main text. Notice that frequencies smaller than 1 T are not considered.

Supplementary Note 1: Derivation of Lifshitz-Kosevich formula

For convenience and completeness of reference, we briefly summarize the derivation of the Lifshitz-Kosevich formula[1] from previous works. We first give the quantization rule of Landau levels. Then an intuitive argument is provided for the final oscillatory part of the grand canonical potential. At last, a formal derivation based on Green's function method is presented.

A. Semiclassical Quantization rule and magnetic oscillation

In the presence of magnetic field B (we assume it's along z direction), the electron will undergo cyclotron motion whose quantization condition gives the quantized Landau level for an arbitrary dispersion. The central requirement for the quantization of orbit is that the phase of wave function accumulated during one cycle must satisfy the interference condition. For now, the phase has mainly six contributions: de Broglie phase, Aharonov-Bohm (A-B) phase, Maslov correction ϕ_M, Berry phase, orbital magnetic moment induced phase and Zeeman (spin) effect induced phase[2–4]. The de Broglie phase is 2π times the number of wavelengths in the orbit $\int \boldsymbol{k} \cdot d\boldsymbol{r}$. It's well-known that the real space trajectory of an electron is the trajectory of k normal to B scaled by magnetic length $l_B = \sqrt{\hbar c/eB}$ and rotated by 90°[5]. The A-B phase is the number of magnetic flux quanta $2\pi\hbar c/e$ through the trajectory multiplied by 2π. For a simple trajectory, it has two caustics when projected onto one direction and each has a $\pi/2$ phase shift similar to the optical phase shift. Finally, there is an additional phase λ_a (a is the band index of degenerate bands) from the k space trajectory which incorporates Berry phase ϕ_B, orbital phase ϕ_R and Zeeman phase ϕ_Z[3,4]. We term it the generalized Berry phase. It can be calculated from the eigenvalue $e^{i\lambda_a}$ of the propagator U$[C]$ of loop C ($\overline{\exp}$ means path-ordered exponential),

$$U[C] = \overline{\exp}\left\{ i \oint \left[(\boldsymbol{A} + \mathfrak{A}) \cdot d\boldsymbol{k} + \frac{g_0 \hbar}{4mv^{\perp}} \sigma^z |d\boldsymbol{k}| \right] \right\} \tag{1}$$

where $\boldsymbol{A}_{mn} = i\langle u_{mk} | \boldsymbol{\nabla}_k u_{nk} \rangle$ ($m, n \in \mathbb{Z}_D$, $\mathbb{Z}_D$ is the degenerate band group being considered.) is the non-Abelian Berry connection and the first term is just the generalization of Berry phase to the multiband case. The second phase which comes from the orbital magnetic moment is

$$\mathfrak{A}_{mn} \cdot d\boldsymbol{k} = \sum_{l \notin \mathbb{Z}_D} A^x_{ml} \Pi^y_{ln} dk_x / 2v_y + (x \leftrightarrow y) \tag{2}$$

with $\boldsymbol{\Pi}_{ln} = \langle u_{lk} | \boldsymbol{v}_{\boldsymbol{k}} | u_{nk} \rangle$ being the interband matrix elements of the group velocity operator $\boldsymbol{v}$. σ^z is the spin Pauli matrix and the third phase comes from the Zeeman effect. g_0 is the Lande g-factor.

The overall interference condition becomes

$$\int \boldsymbol{k} \cdot d\boldsymbol{r} - 2\pi \frac{B l_B^2 A(\varepsilon, k_z)}{2\pi\hbar c/e} - \phi_M + \lambda_a = 2\pi n \tag{3}$$

or

$$l_B^2 A(\varepsilon_n, k_z) = 2\pi(n + \gamma_a) \tag{4}$$

where $A(\varepsilon, k_z)$ is the area of k space trajectory and $\gamma_a = (\phi_M - \lambda_a)/2\pi$ is the index shift of Landau level. This is the Onsager relation with various phases taken into account[3,4,6,7].

Following the argument of Ref. 5, equation (4) specifies a set of Landau tubes (for each n) on which quantized orbits lie. The contribution to the density of states (DOS) $D(\varepsilon_F)$ at the Fermi level ε_F (at zero temperature) from these Landau levels are the number of quantized orbits with energy in a small shell near ε_F. Therefore, $D(\varepsilon_F)$ is proportional to the intersection area between the tubes and the small energy shell around the Fermi surface. When the tube is tangential to the Fermi surface, the area will be greatly enhanced and $D(\varepsilon_F)$ will be singular, i.e.

$$A_e(\varepsilon_F) = (n + \gamma_a) \frac{2\pi e B}{\hbar c} \tag{5}$$

where $A_e(\varepsilon_F)$ is the area of extremal orbit on the Fermi surface. As a consequence, the oscillation of $D(\varepsilon_F)$ is periodic in $\frac{1}{B}$ with period $\Delta\left(\frac{1}{B}\right) = 2\pi e/(\hbar c A_e(\varepsilon_F))$ and frequency

$$F = 1/\Delta\left(\frac{1}{B}\right) = \frac{\hbar c}{2\pi e} A_e(\varepsilon_F) \tag{6}$$

B. Intuitive argument

The oscillation of various thermal quantities such as magnetic susceptibility follows the oscillation of $D(\varepsilon_F)$, or equivalently the grand canonical potential. In general, such an oscillation part $\delta\Omega(T=0,\varepsilon_F)$ can be expanded as a Fourier series with the positions of maximum determined from (5) and fundamental frequency given by (6), i.e.

$$\delta\Omega(T=0,\varepsilon_F) = \sum_r \Omega_r(T=0,\varepsilon_F) = \sum_r c_r \cos[2\pi r(\frac{F}{B}-\gamma_a)+\varphi] = \sum_r c_r \cos[r(l_B^2 A_e(\varepsilon_F)+\lambda_a-\phi_M)+\varphi] \tag{7}$$

where c_r is the amplitude of r'th harmonics and φ is an additional phase shift for for three-dimensional (3D) materials (see below).

The effect of finite temperature and finite relaxation time can be taken into account by the method of phase smearing[8] and contribute a reduction factor for the oscillation amplitude. Here we adopt a different way to get the reduction factor. First, the grand canonical potential at finite T can be obtained from the zero temperature one by convolution with the derivative of the Fermi distribution[8].

$$\delta\Omega(T,\mu) = -\int_{-\infty}^{\infty} \frac{\partial f(\varepsilon-\mu)}{\partial\varepsilon}\delta\Omega(0,\varepsilon)d\varepsilon \tag{8}$$

where $f(\varepsilon-\mu) = 1/[\exp((\varepsilon-\mu)/kT)+1]$ is Fermi-Dirac distribution function and μ is the chemical potential at finite temperature. Substitute (7) into the above formula and make use of the relation (6) to change integral variable from ε to F, one can get

$$\begin{aligned}
\Omega_r(T,\mu) &= -c_r \text{Re}\int_{-\infty}^{\infty} \frac{\partial f(\varepsilon-\mu)}{\partial\varepsilon}\exp[i(2\pi r(\frac{F}{B}-\gamma_a)+\varphi)]d\varepsilon \\
&= c_r \text{Re}\int_{-\infty}^{\infty} \frac{1}{2k_BT[1+\cosh((\varepsilon-\mu)/k_BT)]}\exp[i(2\pi r(\frac{F}{B}-\gamma_a)+\varphi)]d\varepsilon \\
&= c_r \text{Re}\int_{-\infty}^{\infty} \frac{1}{2[1+\cosh((\varepsilon-\mu)/k_BT)]}\exp[i(2\pi r(\frac{F}{B}-\gamma_a)+\varphi)]\frac{2\pi e}{k_BT\hbar c A_e'(\mu)}dF \\
&= c_r \text{Re}\int_{-\infty}^{\infty} \frac{1}{1+\cosh[\beta(F-F(\mu))/k_BT]}\exp[i(2\pi r(\frac{F}{B}-\gamma_a)+\varphi)]\frac{\beta}{2k_BT}dF \\
&= c_r \text{Re}\int_{-\infty}^{\infty} \exp[i(2\pi r(\frac{F(\mu)}{B}-\gamma_a)+\varphi)]\cdot\frac{\exp[i2\pi r k_BTz/(\beta B)]}{2[1+cosh(z)]}dz \\
&= c_r R_{T,r}\cos[2\pi r(\frac{F(\mu)}{B}-\gamma_a)+\varphi] \\
&= R_{T,r}\,\Omega_r(0,\mu)
\end{aligned} \tag{9}$$

with

$$R_{T,r} = \frac{2\pi^2 r k_BT/\beta B}{\sinh(2\pi^2 r k_BT/\beta B)}, \quad \beta = \frac{e\hbar}{mc}, \quad m = \frac{\hbar^2}{2\pi}\frac{\partial A_e(\varepsilon,k_z)}{\partial\varepsilon} \tag{10}$$

m is the cyclotron mass. Use cyclotron frequency $\omega_c = eB/mc$, temperature reduction factor $R_{T,r}$ can also be written as

$$R_{T,r} = \frac{2\pi^2 r k_BT/\hbar\omega_c}{\sinh(2\pi^2 r k_BT/\hbar\omega_c)} \tag{11}$$

Second, a finite relaxation time can be qualitatively considered as the damping of wave function amplitude. The grand canonical potential is proportional to the DOS, which is further proportional to the diagonal elements of Green's function $G(\boldsymbol{r},\boldsymbol{r},E)$. The latter one represents the returning probability amplitude for electron starting from $\boldsymbol{r}$ and returning to $\boldsymbol{r}$. If We assume the wave packets decay in time like[9]

$$|\Psi(t)| = \exp(-t/2\tau) \tag{12}$$

then the oscillation amplitude is reduced by the same factor with t the time for an electron to return to the original position. For r'th harmonics, the time for returning is the time for completing r cycles: $t_r = 2\pi r/\omega_c$ (ω_c is cyclotron frequency). Thus the relaxation time reduction factor (Dingle factor) is[9]

$$R_{D,r} = \exp(-t_r/2\tau) = \exp(-\pi r/\omega_c\tau) \tag{13}$$

Put all these relations together, we get the generalized Lifshitz-Kosevich formula (for one band)[3]

$$\delta\Omega(T,\mu) = \sum_r c_r \frac{2\pi^2 r k_B T/\hbar\omega_c}{\sinh(2\pi^2 l k_B T/\hbar\omega_c)} \exp(-\frac{\pi r}{\omega_c \tau}) \cos[2\pi r(\frac{F}{B} - \gamma_a) + \varphi] \tag{14}$$

C. Formal derivation

For a formal justification of formula (14) and also the determination of the amplitude c_r, we provide a direct calculation of the grand canonical potential taking the finite T and τ into account at the beginning. Here the method is from Champel and Mineev[10]. First, we focus on one band taking possible spin-orbit coupling into consideration (so the wave function is a two-component spinor). In the presence of disorder scattering, if one uses the Born approximation, then the self-energy is

$$\Sigma^R(\varepsilon, k_z) = -i\Gamma = -i\frac{\hbar}{2\tau} \tag{15}$$

where the real part of self-energy is ignored whose effect is only a constant shift of energy in this approximation. τ is the relaxation time used in (12) and gives the Dingle factor as can be seen later. The retarded Green's function is now modified by the self-energy $\Sigma^R(\varepsilon, k_z)$ as

$$G_n^R(\varepsilon, k_z) = \frac{1}{\varepsilon - \varepsilon_n(k_z) - \Sigma^R(\varepsilon, k_z)} \tag{16}$$

where the Landau level $\varepsilon_n(k_z)$ is determined by (4). The spectral density is the imaginary part of Green's function

$$\begin{aligned} S_n(\varepsilon, k_z) &= -\frac{1}{\pi}\mathrm{Im}G_n^R(\varepsilon, k_z) \\ &= \frac{1}{\pi}\frac{\Gamma}{[\varepsilon - \varepsilon_n(k_z)]^2 + \Gamma^2} \end{aligned} \tag{17}$$

The spectral density function can be used to define a generalized density of states $D(\varepsilon)$ whose integral is the particle number. In the present case, each Landau level has a degeneracy $D = eB/2\pi\hbar c$, and $D(\varepsilon)$ is given by

$$D(\varepsilon) = D\int \frac{dk_z}{2\pi} \sum_n S_n(\varepsilon, k_z) \tag{18}$$

To calculate the sum over n, we first define a continuous variable x via

$$A(\varepsilon(x), k_z) = x\frac{2\pi e B}{\hbar c} \tag{19}$$

i.e. $n + \gamma_a$ in (4) is replaced by x. Then with the help of Poisson summation formula[8] ($0 \le \gamma_a \le 1$)

$$\sum_{n=0}^{\infty} f(n+\gamma_a) = \int_0^{+\infty} f(x)dx + 2\sum_{r=1}^{\infty}\int_0^{+\infty} f(x)\cos[2\pi r(x-\gamma_a)]\,dx \tag{20}$$

$D(\varepsilon)$ can be written as

$$D(\varepsilon) = \frac{D}{\pi}\int\frac{dk_z}{2\pi}\left[\int_0^{+\infty} dx \frac{\Gamma}{[\varepsilon - \varepsilon(x,k_z)]^2 + \Gamma^2} + 2\mathrm{Re}\sum_{r=1}^{\infty}\int_0^{+\infty} dx\frac{\Gamma}{[\varepsilon - \varepsilon(x,k_z)]^2 + \Gamma^2}\exp(2\pi i r(x-\gamma_a))\right] \tag{21}$$

The first part of the above formula represents the DOS in the absence of a magnetic field, and the second part is the oscillation part which we shall focus on. Below we calculate the r'th Fourier components in the oscillation part $D_r(\varepsilon)$. Changing the variable from x to $\varepsilon(x, k_z)$ via (19) and use the definition of effective mass $m(\varepsilon)$ (10), the integral is written as

$$D_r(\varepsilon) = \frac{1}{\pi^2\hbar^2}\mathrm{Re}\int \frac{dk_z}{2\pi}\int_{\varepsilon_0}^{+\infty} d\varepsilon' \frac{\Gamma}{[\varepsilon-\varepsilon']^2+\Gamma^2} m(\varepsilon')\exp\left[2\pi i r(\frac{\hbar c}{2\pi eB}A(\varepsilon', k_z)-\gamma_a)\right] \tag{22}$$

The lower integral limit ε_0 is the energy for the cross section area to vanish, i.e., $A(\varepsilon_0, k_z) = 0$. In general, ε_0 depends on k_z, but if we assume $\Gamma << \varepsilon$, then the contribution of the integral over ε' is mainly from the vicinity of ε, hence the lower integral limit can be extended to $-\infty$. Then interchange the order of integral and do the integral over k_z first, we need to calculate

$$\int \frac{dk_z}{2\pi}\exp\left[2\pi i r(\frac{\hbar c}{2\pi eB}A(\varepsilon', k_z)-\gamma_a)\right] \tag{23}$$

This integral can be done by using the stationary phase approximation. Physically, the integral is a superposition of oscillation of DOS from all different k_z. If the cross-section area $A(\varepsilon', k_z)$ changes rapidly, then these contributions cancel out. The only exception is the extremal orbit where $dA(\varepsilon', k_z)/dk_z$ vanishes and the oscillation from the orbits around it adds coherently. Hence, after integrating over k_z, only the extremal orbit has an appreciable contribution. The result is simply

$$\sqrt{\frac{eB}{2\pi\hbar cr|A_e''|}}\exp\left[2\pi i r(\frac{\hbar c}{2\pi eB}A_e(\varepsilon')-\gamma_a)\pm i\frac{\pi}{4}\right] \tag{24}$$

where $A_e(\varepsilon')$ is the area of extremal orbit and there is an additional phase shift $\pm\frac{\pi}{4}$ (+ for minimum area and − for maximum area). Substitute this result into (22), and complete the integral, the r'th component is found as

$$\begin{aligned} D_r(\varepsilon) &= \frac{1}{\pi^2\hbar^2}\sqrt{\frac{eB}{2\pi\hbar cr|A_e''|}}\mathrm{Re}\int_{-\infty}^{+\infty} d\varepsilon' \frac{\Gamma}{[\varepsilon-\varepsilon']^2+\Gamma^2} m(\varepsilon')\exp\left[2\pi i r(\frac{\hbar c}{2\pi eB}A_e(\varepsilon')-\gamma_a)\pm i\frac{\pi}{4}\right] \\ &\approx \frac{m(\varepsilon)}{\pi^2\hbar^2}\sqrt{\frac{eB}{2\pi\hbar cr|A_e''|}}\mathrm{Re}\exp\left[2\pi i r(\frac{\hbar c}{2\pi eB}A_e(\varepsilon)-\gamma_a)\pm i\frac{\pi}{4}\right]\int_{-\infty}^{+\infty} d\varepsilon' \frac{\Gamma}{[\varepsilon'-\varepsilon]^2+\Gamma^2}\exp\left[i\frac{r\hbar c}{eB}A_e'(\varepsilon)(\varepsilon'-\varepsilon)\right] \\ &= \frac{m(\varepsilon)}{\pi^2\hbar^2}\sqrt{\frac{eB}{2\pi\hbar cr|A_e''|}}\mathrm{Re}\exp\left[2\pi i r(\frac{\hbar c}{2\pi eB}A_e(\varepsilon)-\gamma_a)\pm i\frac{\pi}{4}\right]\int_{-\infty}^{+\infty} dy \frac{\Gamma}{y^2+\Gamma^2}\exp\left[i\frac{2\pi r}{\hbar\omega_c}y\right] \\ &= \frac{m(\varepsilon)}{\pi^2\hbar^2}\sqrt{\frac{eB}{2\pi\hbar cr|A_e''|}}\mathrm{Re}\exp\left[2\pi i r(\frac{\hbar c}{2\pi eB}A_e(\varepsilon)-\gamma_a)\pm i\frac{\pi}{4}\right] 2\pi i\cdot\frac{\Gamma\exp(-\frac{2\pi r\Gamma}{\hbar\omega_c})}{2i\Gamma} \\ &= (\frac{eB}{2\hbar c})^{1/2}\frac{m(\varepsilon)}{\pi^{3/2}\hbar^2\sqrt{r|A_e''|}}\cos\left[2\pi r(\frac{F}{B}-\gamma_a)\pm\frac{\pi}{4}\right]\exp(-\frac{\pi r}{\omega_c\tau}) \end{aligned} \tag{25}$$

where ω_c is cyclotron frequency of the extremal orbit with energy ε and $F = \hbar cA_e(\varepsilon)/2\pi e$. The last exponential factor is just the Dingle factor (13).

To proceed, the particle number at chemical potential μ (at finite temperature) is $N(\mu) = \int D(\varepsilon)f(\varepsilon-\mu)d\varepsilon$, and use thermal dynamic relation $N = -\frac{\partial\Omega(T,\mu)}{\partial\mu}|_T$. The r'th component of grand canonical potential (per unit volume) is

$$\begin{aligned} \frac{\Omega_r(T,\mu)}{V} &= -\int_{-\infty}^{\mu} d\zeta\int_{-\infty}^{+\infty} d\varepsilon D_r(\varepsilon)f(\varepsilon-\zeta) \\ &= -k_BT\int_{-\infty}^{+\infty} d\varepsilon D_r(\varepsilon)\ln\{1+\exp[-(\varepsilon-\mu)/k_BT]\} \\ &= -(\frac{eB}{2\hbar c})^{1/2}\frac{m}{\pi^{3/2}\hbar^2\sqrt{r|A_e''|}}\exp(-\frac{\pi r}{\omega_c\tau})k_BT\,\mathrm{Re}\int_{-\infty}^{+\infty} d\varepsilon\exp\left[2\pi i r(\frac{F(\varepsilon)}{B}-\gamma_a)\pm i\frac{\pi}{4}\right]\ln\{1+\exp[-(\varepsilon-\mu)/k_BT]\} \end{aligned} \tag{26}$$

In the above formula, all the prefactors are evaluated at μ since most contribution comes from the Fermi surface. The integral is done by integration by parts twice and evaluate $F'(\varepsilon)$ at chemical potential $F'(\mu) = \beta^{-1} = B/\hbar\omega_c$ and expand $F(\varepsilon)$ around μ.

$$
\begin{aligned}
&k_BT\int_{-\infty}^{+\infty}d\varepsilon\exp\left[2\pi ir(\frac{F(\varepsilon)}{B}-\gamma_a)\pm i\frac{\pi}{4})\right]\ln\{1+\exp\left[-(\varepsilon-\mu)/k_BT\right]\}\\
=&k_BT(\frac{\hbar\omega_c}{2\pi ir})^2(-\frac{1}{k_BT})^2\frac{1}{2}\int_{-\infty}^{+\infty}d\varepsilon\frac{\exp\left[2\pi ir(\frac{F(\varepsilon)}{B}-\gamma_a)\pm i\frac{\pi}{4}\right]}{1+\cosh\left[(\varepsilon-\mu)/k_BT\right]}\\
=&-(\frac{\hbar\omega_c}{2\pi r})^2\exp\left[2\pi ir(\frac{F(\mu)}{B}-\gamma_a)\pm i\frac{\pi}{4}\right]\frac{1}{2k_BT}\int_{-\infty}^{+\infty}d\varepsilon\frac{\exp\left[2\pi ir(\varepsilon-\mu)/\hbar\omega_c\right]}{1+\cosh\left[(\varepsilon-\mu)/k_BT\right]}\\
=&-(\frac{\hbar\omega_c}{2\pi r})^2\exp\left[2\pi ir(\frac{F(\mu)}{B}-\gamma_a)\pm i\frac{\pi}{4}\right]R_{T,r}
\end{aligned}
\tag{27}
$$

Hence,

$$
\frac{\Omega_r(T,\mu)}{V}=\frac{\hbar\omega_c}{(2\pi l_B^2)^{3/2}}\frac{1}{2\pi^2 r^{5/2}\sqrt{|A_e''|}}\cdot\cos\left[2\pi r(\frac{F(\mu)}{B}-\gamma_a)\pm\frac{\pi}{4}\right]R_{T,r}R_{D,r}
\tag{28}
$$

This is the same as (14) and gives the explicit expression for amplitude c_r and phase shift φ, which verifies our previous intuitive argument 3D system. The oscillatory part of total grand canonical potential $\delta\Omega(T,\mu)$ is the sum of Ω_r over Fourier components r and degenerated bands a. The magnetization can be obtained by differentiating with B in the cosine factor (this is the dominant part): $M=-1/V(\partial\Omega/\partial B)$.

$$
\begin{aligned}
\frac{\delta\Omega(T,\mu)}{V}&=\sum_a\sum_r\frac{\hbar\omega_c}{(2\pi l_B^2)^{3/2}}\frac{1}{2\pi^2 r^{5/2}\sqrt{|A_e''|}}\cos\left[2\pi r(\frac{F}{B}-\gamma_a)\pm\frac{\pi}{4}\right]R_{T,r}R_{D,r}\\
&=\sum_a\sum_r\frac{\hbar\omega_c}{(2\pi l_B^2)^{3/2}}\frac{1}{2\pi^2 r^{5/2}\sqrt{|A_e''|}}\cos\left[r(l_B^2A_e+\lambda_a-\phi_M)\pm\frac{\pi}{4}\right]R_{T,r}R_{D,r}
\end{aligned}
\tag{29}
$$

$$
\begin{aligned}
M(T,\mu)&=-\sum_a\sum_r\frac{\hbar\omega_c}{(2\pi l_B^2)^{3/2}}\frac{F}{\pi r^{3/2}B^2\sqrt{|A_e''|}}\sin\left[2\pi r(\frac{F}{B}-\gamma_a)\pm\frac{\pi}{4}\right]R_{T,r}R_{D,r}\\
&=-\sum_a\sum_r\frac{\hbar\omega_c}{(2\pi l_B^2)^{3/2}}\frac{F}{\pi r^{3/2}B^2\sqrt{|A_e''|}}\sin\left[r(l_B^2A_e+\lambda_a-\phi_M)\pm\frac{\pi}{4}\right]R_{T,r}R_{D,r}
\end{aligned}
\tag{30}
$$

F takes value at μ and $R_{T,r}, R_{D,r}$ are given by (11), (13) respectively.

If the spin-orbital coupling (SOC) is very weak or zero, each band degenerates for two spin channels (one band with two electrons). There is only one Berry phase ϕ_B, one orbital phase ϕ_R and two opposite Zeeman phases ϕ_Z. Then the generalized Berry phase λ_a can be factorized into two parts: $\tilde{\lambda}=\phi_B+\phi_R$ and $\pm\phi_Z$, whose values can be calculated by, according to Eq. (1),

$$
\phi_B=\oint \boldsymbol{A}\cdot d\boldsymbol{k},\qquad \phi_R=\oint \mathfrak{A}\cdot d\boldsymbol{k},\qquad \phi_Z=\oint\frac{g_0\hbar}{4mv^{\perp}}d\boldsymbol{k}=\frac{\pi g_0 m}{2m_0}.
\tag{31}
$$

Now the total grand canonical potential of one band (degenerate with respect to spin) is

$$
\begin{aligned}
\frac{\delta\tilde{\Omega}(T,\mu)}{V}&=\sum_r\frac{\hbar\omega_c}{(2\pi l_B^2)^{3/2}}\frac{1}{2\pi^2 r^{5/2}\sqrt{|A_e''|}}\left\{\cos\left[r(l_B^2A_e+\tilde{\lambda}+\phi_Z-\phi_M)\pm\frac{\pi}{4}\right]+\cos\left[r(l_B^2A_e+\tilde{\lambda}-\phi_Z-\phi_M)\pm\frac{\pi}{4}\right]\right\}R_{T,r}R_{D,r}\\
&=\sum_r\frac{\hbar\omega_c}{(2\pi l_B^2)^{3/2}}\frac{1}{\pi^2 r^{5/2}\sqrt{|A_e''|}}\cos\left[r(l_B^2A_e+\tilde{\lambda}-\phi_M)\pm\frac{\pi}{4}\right]R_{T,r}R_{D,r}R_{s,r}
\end{aligned}
\tag{32}
$$

where

$$
R_{s,r}=\cos(r\phi_Z)=\cos(\frac{r\pi g_0 m}{2m_0})
\tag{33}
$$

is the spin reduction factor[1,8]. One important difference between R_s and R_T, R_D is that the latter is always positive while the former can be negative. The negativity of R_s for the first harmonic means an additional π phase shift in the oscillation peaks and is usually considered separately to extract the phase $\tilde{\lambda}$. If the system has further inversion and time-reversal symmetries, the Berry phase ϕ_B can only be 0 or π and the orbital phase ϕ_R can only be zero. The final phase shift will be determined easily by ϕ_B and cyclotron mass m that determines the sign of R_s. In detail, if $\phi_B = \pi$ and m is small ($R_s > 0$), the phase shift is π. This is the case with graphene. If $\phi_B = 0$ and m makes $R_s < 0$, then phase shift is also π. All other combinations lead to no extra phase shift in quantum oscillation.

If SOC is strong, the wave function for each band is a two-component spinor, so the separation of a spin reduction factor is no longer possible. Further, if all bands are degenerate, λ_a must be calculated as a whole to ensure gauge invariant results. In the case of spin degeneracy ($a = 1, 2$), the sum of two fundamental harmonics is still a harmonic with the same frequency but reduced amplitude

$$\cos\left[r(l_B^2 A_e + \lambda_1 - \phi_M) \pm \frac{\pi}{4}\right] + \cos\left[r(l_B^2 A_e + \lambda_2 - \phi_M) \pm \frac{\pi}{4}\right] = 2|\cos(\frac{\lambda_1 - \lambda_2}{2})| \cdot \cos\left[r(l_B^2 A_e + \Delta\phi - \phi_M) \pm \frac{\pi}{4}\right] \tag{34}$$

where

$$\Delta\phi = \frac{\lambda_1 + \lambda_2}{2} + \pi\left\{1 - \eta\left[\cos(\frac{\lambda_1 - \lambda_2}{2})\right]\right\} \tag{35}$$

with $\eta(x)$ the Heaviside step function. In the general case, $|\cos(\frac{\lambda_1 - \lambda_2}{2})|$ is a generalization of spin reduction factor and $\Delta\phi$ determines the extra phase shift of the oscillation peak position in first harmonic. In the case of both inversion and time-reversal symmetries, $\lambda_1 = -\lambda_2 = \lambda$. The final phase shift $\Delta\phi$ is 0 if $\cos(\lambda)$ is positive ($|\lambda| < 0.5\pi$) and π if $\cos(\lambda)$ is negative ($|\lambda| > 0.5\pi$). If $|\lambda| = 0.5\pi$, the oscillation may not observable because $\cos(\lambda) = 0$.

In 2D systems, the particle number is fixed while chemical potential oscillates around $\mu(B = 0)$ with an appreciable amplitude. This oscillation causes a change of the frequency F and the resulting oscillatory behavior is different from 3D cases[11]. But if $\mu >> \hbar\omega_c$ and the reduction factor is small enough, then the oscillation of μ can be ignored and similar results are obtained[10]. It can be calculated by the same method above, where the integration over k_z is omitted (hence, the phase $\pm\pi/4$ disappears). Here, we only give the final expression for the oscillation part of grand canonical potential and magnetization

$$\begin{aligned} \frac{\delta\Omega(T,\mu)}{S} &= \sum_a \sum_r \frac{\hbar\omega_c}{2\pi l_B^2} \frac{1}{\pi^2 r^2} \cos\left[2\pi r(\frac{F}{B} - \gamma_a)\right] R_{T,r} R_{D,r} \\ &= \sum_a \sum_r \frac{\hbar\omega_c}{2\pi l_B^2} \frac{1}{\pi^2 r^2} \cos\left[r(l_B^2 A_e + \lambda_a - \phi_M)\right] R_{T,r} R_{D,r} \qquad (2D) \end{aligned} \tag{36}$$

$$\begin{aligned} M(T,\mu) &= -\sum_a \sum_r \frac{\hbar\omega_c}{2\pi l_B^2} \frac{2F}{\pi r B^2} \sin\left[2\pi r(\frac{F}{B} - \gamma_a)\right] R_{T,r} R_{D,r} \\ &= -\sum_a \sum_r \frac{\hbar\omega_c}{2\pi l_B^2} \frac{2F}{\pi r B^2} \sin\left[r(l_B^2 A_e + \lambda_a - \phi_M)\right] R_{T,r} R_{D,r} \qquad (2D) \end{aligned} \tag{37}$$

where S is the area of material and reduction factors $R_{T,r}, R_{D,r}$ are the same as before.

Finally, we point out that if one considers higher order terms in the phase γ_a (the effective Hamiltonian is expanded to second order in B), γ_a can depend on the field B[12]. If $\gamma_a = \gamma_{a0} + \gamma_{a1}B$ and $\gamma_{a1} << 1/B$, then such a field dependence can be regarded as the change of oscillation frequency with the magnetic field, $F(B) = F(0) - \gamma_{a1}B^2$.

Supplementary Note 2: Berry phase in time reversal and inversion symmetric system

In this section, we discuss the Berry phase in a system with intrinsic spin-orbit coupling, which preserves both time-reversal (TRS) $\mathcal{T}$ and inversion symmetries (IS) $\mathcal{P}$. The kagome lattice model used in the main text has such properties and the analysis here can be directly applied to it. Due to $\mathcal{PT}$ symmetry, every energy band is at least two-fold degenerate so can be labeled by band index n and pseudospin index s $(= \pm 1)$. Under time reversal operation, the band index remains the same but pseudospin index s is changed to each other ($s = 1 \leftrightarrow s = -1$).

First, consider the effect of $\mathcal{T}$. Under time reversal, Bloch states flip pseudospin and momentum but has the same energy (same band index n)

$$\mathcal{T}|u_{n1}(\boldsymbol{k})\rangle = |u_{n-1}(-\boldsymbol{k})\rangle \tag{38}$$

Therefore, the Berry connection for different pseudospin components has the relation

$$\boldsymbol{A}_{n-1}(-\boldsymbol{k}) = i\langle u_{n-1}(-\boldsymbol{k})|\nabla_{-k}|u_{n-1}(-\boldsymbol{k})\rangle = -i\langle \mathcal{T}u_{n1}(\boldsymbol{k})|\nabla_k|\mathcal{T}u_{n1}(\boldsymbol{k})\rangle = -i\langle \nabla_k u_{n1}(\boldsymbol{k})|u_{n1}(\boldsymbol{k})\rangle = i\langle u_{n1}(\boldsymbol{k})|\nabla_k|u_{n1}(\boldsymbol{k})\rangle = \boldsymbol{A}_{n1}(\boldsymbol{k}) \tag{39}$$

The Berry phase along the loop C for one pseudospin is therefore opposite to the Berry phase of the inversion loop $\overline{C}$ for opposite pseudospin

$$\phi_{n1}(C) = \int_C \boldsymbol{A}_{n1}(\boldsymbol{k}) \cdot d\boldsymbol{k} = \int_C \boldsymbol{A}_{n-1}(-\boldsymbol{k}) \cdot d\boldsymbol{k} = -\int_C \boldsymbol{A}_{n-1}(-\boldsymbol{k}) \cdot d(-\boldsymbol{k}) = -\int_{\overline{C}} \boldsymbol{A}_{n-1}(\boldsymbol{k}) \cdot d\boldsymbol{k} = -\phi_{n-1}(\overline{C}) \tag{40}$$

Next, we consider the role of inversion symmetry. Under inversion operation, Bloch states reverse momentum but preserves pseudospin and energy

$$\mathcal{P}|u_{ns}(\boldsymbol{k})\rangle = |u_{ns}(-\boldsymbol{k})\rangle \tag{41}$$

The Berry connection for the same pseudospin at opposite positions has the relation

$$\boldsymbol{A}_{ns}(-\boldsymbol{k}) = i\langle u_{ns}(-\boldsymbol{k})|\nabla_{-k}|u_{ns}(-\boldsymbol{k})\rangle = -i\langle \mathcal{P}u_{ns}(\boldsymbol{k})|\nabla_k|\mathcal{P}u_{ns}(\boldsymbol{k})\rangle = -i\langle u_{ns}(\boldsymbol{k})|\nabla_k|u_{ns}(\boldsymbol{k})\rangle = -\boldsymbol{A}_{ns}(\boldsymbol{k}) \tag{42}$$

So the Berry phase along the loop C for one pseudospin now is the same for the inversion loop $\overline{C}$ with the same pseudospin

$$\phi_{ns}(C) = \int_C \boldsymbol{A}_{ns}(\boldsymbol{k}) \cdot d\boldsymbol{k} = -\int_C \boldsymbol{A}_{ns}(-\boldsymbol{k}) \cdot d\boldsymbol{k} = \int_C \boldsymbol{A}_{ns}(-\boldsymbol{k}) \cdot d(-\boldsymbol{k}) = \int_{\overline{C}} \boldsymbol{A}_{ns}(\boldsymbol{k}) \cdot d\boldsymbol{k} = \phi_{ns}(\overline{C}) \tag{43}$$

If the system has both $\mathcal{T}$ and $\mathcal{P}$, combining the results above, we obtain that Berry phases around the same loop for different pseudospin components are opposite

$$\begin{aligned} \boldsymbol{A}_{ns}(\boldsymbol{k}) &= \boldsymbol{A}_{n-s}(-\boldsymbol{k}) = -\boldsymbol{A}_{n-s}(\boldsymbol{k}) \\ \phi_{ns}(C) &= -\phi_{n-s}(\overline{C}) = -\phi_{n-s}(C) \end{aligned} \tag{44}$$

The Berry phase with spin taken into consideration is very different from that of the spinless case. For example, consider a Fermi surface around the Brillouin zone center (Γ point, Fig. 6) and assume only time-reversal symmetry. In the spinless case, band energy satisfies $\varepsilon(\boldsymbol{k}) = \varepsilon(-\boldsymbol{k})$, the trajectory of such a Fermi surface must be symmetric with respect to Γ, i.e., $C = \overline{C}$. Equations (39) and (40) become

$$\begin{aligned} \boldsymbol{A}_n(\boldsymbol{k}) &= \boldsymbol{A}_n(-\boldsymbol{k}) \\ \phi_n(C) &= -\phi_n(\overline{C}) = 0 \end{aligned} \tag{45}$$

The Berry phase around Γ point must be zero. This argument is also applied to Fermi surfaces surrounding any other time-reversal invariant momenta. But if the spin is added, then one can only draw the conclusion from (40) that Berry phases of two pseudospins with $\mathcal{T}$-correlated loops C and $\overline{C}$ are opposite.

Finally, for a system with both $\mathcal{T}$ and $\mathcal{P}$, not only the Berry phases for the two pseudospin channels with the same loop C (44) are opposite, but also the total phases (i.e., the generalized Berry phase λ_a, composed of Berry phase, orbital phase, and Zeeman phase) of two pseudospin channels are opposite[3]. As a result, the extra phase shift $\Delta\phi$ (35) in the quantum oscillation can only be 0 or π, depending on whether λ_a is smaller or larger than 0.5π. Hence, in general, the π phase determined from the oscillation peak position can't serve as evidence of a Dirac point but may solely come from the system's symmetry[3].

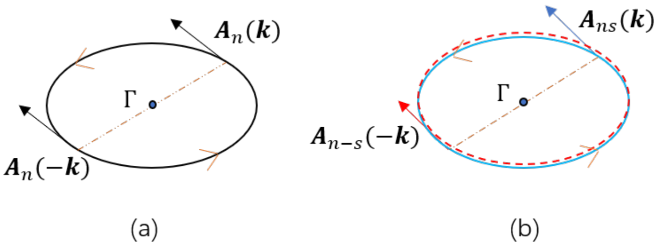

Supplementary Figure 6. The Berry connection for the loop around Γ point, (a) for a spinless case whose Berry phase is zero, and (b) for a spinful case with two spin components. The blue line represents spin s and the red line represents spin $-s$. Their Berry phases are opposite to each other.

Supplementary Note 3: Berry phase and orbital phase in Dirac and BHZ model

First, we apply the above results to the 2D massive Dirac model. It can describe graphene with SOC taking into account or the cross section in k space of Weyl semimetal if the mass is zero. Since the band gap opens at the Dirac point, the Berry phase is usually no longer π. But the orbital moment phase can compensate the Berry phase and their sum can be π independent of the mass Δ of Dirac fermion[2].

To be more precise, consider the following two band Dirac Hamiltonian with Semenoff mass Δ

$$H_s(k) = \hbar v_F(k_x\sigma_x + k_y\sigma_y) + \Delta\sigma_z \tag{46}$$

In the graphene case, $\Delta = s\lambda_{SO}$ with $\lambda_{SO} > 0$ represents SOC strength and $s = \pm 1$ represents different spin indices. The Hamiltonian gives rise two bands with energy $\pm\varepsilon(\boldsymbol{k}) = \pm\sqrt{\hbar^2 v_F^2 \boldsymbol{k}^2 + \Delta^2}$. Since band structure is non-degenerate, three contributions to λ_a can be calculated separately. The Berry phase of the conduction band for a loop with Fermi wave vector k_F around the Dirac point is

$$\phi_B(k_F) = \int_{|\boldsymbol{k}|=k_F} \boldsymbol{A}(\boldsymbol{k}) \cdot d\boldsymbol{k} = \int_{|\boldsymbol{k}|<k_F} dk_x dk_y F_{xy}(\boldsymbol{k}) = -\pi(1 - \frac{\Delta}{\varepsilon(k_F)}) \tag{47}$$

where $F_{xy} = -\Delta/(\hbar^2 v_F^2 k_F^2 + \Delta^2)^{3/2}$ is the Berry curvature Ω. The Berry phase is near zero at the band bottom and approaches $-\pi$ when $E_F = \varepsilon(k_F)$ is much larger than the gap Δ. In this two-band model with particle-hole symmetry, the orbital magnetic moment is proportional to Berry curvature $M(\boldsymbol{k}) = [e/(\hbar c)]\,\varepsilon(\boldsymbol{k})\Omega$. Hence, the orbital moment phase (2) is

$$\phi_R(k_F) = l_B^2 \oint MB\frac{|d\boldsymbol{k}|}{v^{\perp}} = \oint \varepsilon\Omega\frac{|d\boldsymbol{k}|}{v^{\perp}} = \varepsilon\frac{d\phi_B}{d\varepsilon} \tag{48}$$

where $v^{\perp}$ is the group velocity component perpendicular to B. If the Zeeman phase ϕ_Z can be ignored or extracted as a spin reduction factor, λ_a is $-\pi$ independent of Δ.

$$\phi_B + \phi_R = \phi_B + \varepsilon\frac{d\phi_B}{d\varepsilon} = \frac{d(\varepsilon\phi_B)}{d\varepsilon} = -\pi \tag{49}$$

The numerically calculated Berry phase ϕ_B and the phase sum $\phi_B + \phi_R$ are shown in Fig. 7, which is consistent with (49). Hence, even if the Dirac crossing is gapped by SOC, the oscillation phase can still manifest itself as π. It can be attributed to the pure Berry phase only when the mass is zero. In the case of Graphene, the SOC gap Δ is much smaller than typical Fermi energy, which leads to an almost zero ϕ_R and π Berry phase (the far right of Fig. 7 (b)).

Next, we consider the 3D BHZ model which represents the SOC-induced band anti-crossing

$$H(\mathbf{k}) = \epsilon(\mathbf{k})\cdot I + A(k_x\sigma_x s_z - k_y\sigma_y) + M(\mathbf{k})\sigma_z + \tilde{A}k_z\sigma_x s_x = \epsilon(\mathbf{k})\cdot I + \begin{pmatrix} M(\mathbf{k}) & Ak_+ & 0 & \tilde{A}k_z \\ Ak_- & -M(\mathbf{k}) & \tilde{A}k_z & 0 \\ 0 & \tilde{A}k_z & M(\mathbf{k}) & -Ak_- \\ \tilde{A}k_z & 0 & -Ak_+ & -M(\mathbf{k}) \end{pmatrix} \tag{50}$$

where $\epsilon(\mathbf{k})$ contributes only to the band energy and can be set as zero. $k_\pm = k_x \pm ik_y$ and $M(\mathbf{k}) = M_0 - B(k_x^2 + k_y^2 + k_z^2)$ is a k_z dependent mass. This model is a trivial insulator when $M_0/B < 0$ while is a $\mathbb{Z}_2$ topological insulator when $M_0/B > 0$. It has several important symmetries: time reversal and inversion symmetry which ensure energy bands are two-fold degenerate, particle-hole symmetry, and mirror symmetry with respect to $k_z = 0$ plane. The last two symmetries can be mathematically represented as

$$(\sigma_x s_z)H(\mathbf{k})(\sigma_x s_z)^{-1} = -H(\mathbf{k}) \qquad s_z H(\mathbf{k}^{\perp}, k_z)s_z^{-1} = H(\mathbf{k}^{\perp}, -k_z) \tag{51}$$

Due to the mirror symmetry, the extremal cyclotron orbit lies on the $k_z = 0$ plane and we focus on this orbit in the conduction band. This orbit is a circle around Γ point with Fermi wave vector k_F and has two-fold degeneracy. The band energy in this mirror symmetry plane and the magnetic oscillation phase associated with this extremal orbit are shown in Fig. 7, which has an opposite magnetic oscillation phase for degenerate bands due to $\mathcal{PT}$ symmetry. The Berry phase grows continuously from 0 to 2π while the phase sum $\phi_B + \phi_R$ decreases from π to 0 as k_F is larger. When the energy is close to the band edge, the total oscillation phase is close to π if spin contribution ϕ_Z can be ignored. However, this phase originates solely from the orbital moment and the Berry phase is nearly zero instead.

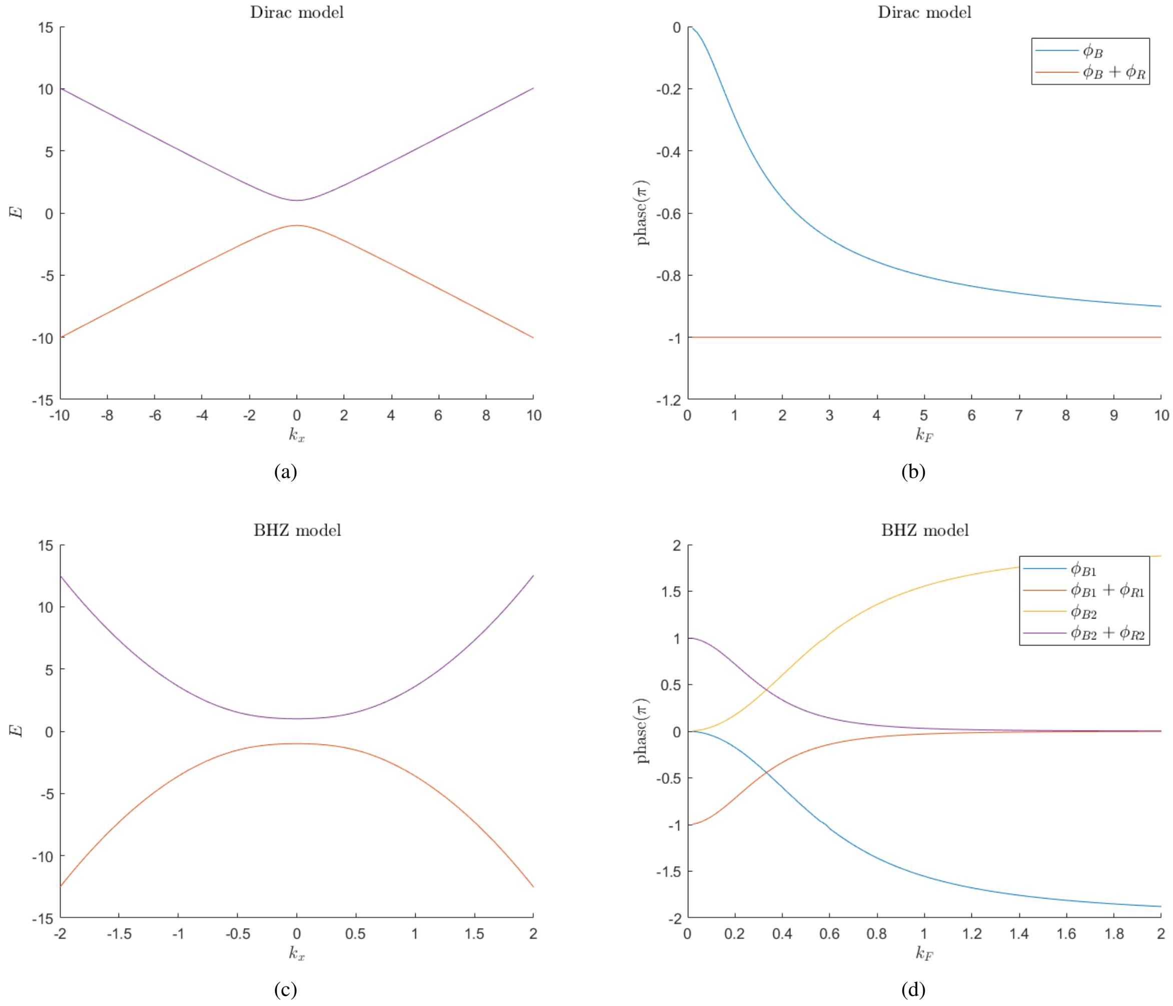

Supplementary Figure 7. (a) Band energy and (b) Berry phase and the phase sum of gapped Dirac model (46). The parameters are $\hbar v = 1$ and Semenoff mass $\Delta = 1$. (b) Band energy in $k_z = 0$ mirror plane and (c) Berry phase and the phase sum of BHZ model (50). The parameters are $A = B = 3$ and $M_0 = \tilde{A} = 1$ which is in $\mathbb{Z}_2$ topological insulator region.

Supplementary Table 1. Berry phase ϕ_B, orbital phase ϕ_R and Zeeman phase ϕ_Z of quantum orbits of 2×2×2 CDW at −40 meV calculated by using the expression (1). Under SOC, the orbital and spin magnetic moment-related phases can only be calculated together with the Berry phase, and (ϕ_B+ϕ_R+ϕ_Z) is the generalized Berry phase (λ) in the main text. Frequency (Freq.) is unit of T. All phases are in unit of π. For the 912 T, we could not get converged results for ϕ_B+ϕ_R and ϕ_B+ϕ_R+ϕ_Z. Notice that the numerical convergence of λ_a for the quantum orbit of 912 T at –40 meV is not as good as others.

Frequency	no SOC			SOC		
	ϕ_B	ϕ_R	ϕ_Z	ϕ_B	ϕ_B+ϕ_R	ϕ_B+ϕ_R+ϕ_Z
13	1	0	0.27	0.83	0.92	0.68
20	0	0	0.44	0.00	0.04	0.38
30	1	0	0.30	0.85	0.83	0.83
77	0	0	0.68	0.51	0.35	0.92
98	0	0	0.45	0.36	0.49	0.87
152	0	0	0.57	0.14	0.60	0.98
202	0	0	0.83	0.56	0.53	0.37
230	0	0	0.50	0.51	0.54	0.84
609	0	0	0.30	0.17	0.18	0.48
730	0	0	0.38	0.63	0.66	0.92
734	0	0	0.22	0.90	0.33	0.22
894	0	0	0.47	0.61	0.66	0.19
898	0	0	0.42	0.23	0.34	0.71
912	0	0	0.22	0.45	0.97	0.31
1067	0	0	0.89	0.06	0.82	0.33
1445	0	0	0.50	0.79	0.60	0.26

Supplementary Table 2. Similar to Table 1 but at –85 meV.

Frequency	no SOC			SOC		
	ϕ_B	ϕ_R	ϕ_Z	ϕ_B	ϕ_B+ϕ_R	ϕ_B+ϕ_R+ϕ_Z
5				0.14	0.79	0.93
14	1	0	0.26	0.99	0.98	0.77
33	0	0	0.23	0.85	0.59	0.84
40	0	0	0.15	0.82	0.64	0.44
51	0	0	0.21	0.65	0.34	0.52
76	0	0	0.33	0.72	0.25	0.02
99	0	0	0.49	0.72	0.62	0.89
224	0	0	0.64	0.01	0.07	0.57
429	0	0	0.59	0.57	0.34	0.06
605	1	0	0.70	0.98	0.92	0.38
617	1	0	0.71	0.99	0.93	0.35
672	0	0	0.25	0.76	0.02	0.11
735	0	0	0.35	0.18	0.20	0.55
765	0	0	0.93	0.71	0.08	0.60
940	0	0	0.48	0.30	0.53	0.07
1267	0	0	0.76	0.84	0.98	0.25
1293	0	0	0.03	0.67	0.57	0.08

Supplementary Note 4: Supplementary Tables

Supplementary references

[1] I. M. Lifshitz and A. M. Kosevich, "Theory of magnetic susceptibility in metals at low temperatures," Sov. Phys. JETP **2**, 636 (1956).
[2] J. N. Fuchs, F. Piéchon, M. O. Goerbig, and G. Montambaux, "Topological berry phase and semiclassical quantization of cyclotron orbits for two dimensional electrons in coupled band models," The European Physical Journal B **77**, 351–362 (2010).
[3] A. Alexandradinata, Chong Wang, Wenhui Duan, and Leonid Glazman, "Revealing the topology of fermi-surface wave functions from magnetic quantum oscillations," Phys. Rev. X **8**, 011027 (2018).
[4] A. Alexandradinata and Leonid Glazman, "Semiclassical theory of landau levels and magnetic breakdown in topological metals," Phys. Rev. B **97**, 144422 (2018).
[5] N. W. Ashcroft and N. D. Mermin, Solid State Physics (Holt-Saunders, 1976).
[6] Ganesh Sundaram and Qian Niu, "Wave-packet dynamics in slowly perturbed crystals: Gradient corrections and berry-phase effects," Phys. Rev. B **59**, 14915–14925 (1999).
[7] G. P. Mikitik and Yu. V. Sharlai, "Manifestation of berry's phase in metal physics," Phys. Rev. Lett. **82**, 2147 (1999).
[8] D. Shoenberg, Magnetic Oscillations in Metals, Cambridge Monographs on Physics (Cambridge University Press, 1984).
[9] L. M. Falicov and Henryk Stachowiak, "Theory of the de haas-van alphen effect in a system of coupled orbits. application to magnesium," Phys. Rev. **147**, 505–515 (1966).
[10] T. Champel and V. P. Mineev, "de haas–van alphen effect in two- and quasi-two-dimensional metals and superconductors," Philosophical Magazine B **81**, 55–74 (2001).
[11] Thierry Champel, "Chemical potential oscillations and de haas–van alphen effect," Phys. Rev. B **64**, 054407 (2001).
[12] Laura M. Roth, "Semiclassical theory of magnetic energy levels and magnetic susceptibility of bloch electrons," Phys. Rev. **145**, 434–448 (1966).
